# Toward Rigorous Parameterization of Underconstrained Neural Network Models Through Interactive Visualization and Steering of Connectivity Generation

**DOI:** 10.3389/fninf.2018.00032

**Published:** 2018-06-01

**Authors:** Christian Nowke, Sandra Diaz-Pier, Benjamin Weyers, Bernd Hentschel, Abigail Morrison, Torsten W. Kuhlen, Alexander Peyser

**Affiliations:** ^1^Visual Computing Institute, RWTH Aachen University, JARA-HPC, Aachen, Germany; ^2^SimLab Neuroscience, Jülich Supercomputing Centre (JSC), Institute for Advanced Simulation, JARA, Forschungszentrum Jülich GmbH, Jülich, Germany; ^3^Institute of Neuroscience and Medicine, Institute for Advanced Simulation, JARA Institute Brain Structure-Function Relationships, Forschungszentrum Jülich GmbH, Jülich, Germany; ^4^Institute of Cognitive Neuroscience, Faculty of Psychology, Ruhr-University Bochum, Bochum, Germany

**Keywords:** simulation and modeling, neural networks, structural plasticity, interactive systems, high performance computing, visualization software

## Abstract

Simulation models in many scientific fields can have non-unique solutions or unique solutions which can be difficult to find. Moreover, in evolving systems, unique final state solutions can be reached by multiple different trajectories. Neuroscience is no exception. Often, neural network models are subject to parameter fitting to obtain desirable output comparable to experimental data. Parameter fitting without sufficient constraints and a systematic exploration of the possible solution space can lead to conclusions valid only around local minima or around non-minima. To address this issue, we have developed an interactive tool for visualizing and steering parameters in neural network simulation models. In this work, we focus particularly on connectivity generation, since finding suitable connectivity configurations for neural network models constitutes a complex parameter search scenario. The development of the tool has been guided by several use cases—the tool allows researchers to steer the parameters of the connectivity generation during the simulation, thus quickly growing networks composed of multiple populations with a targeted mean activity. The flexibility of the software allows scientists to explore other connectivity and neuron variables apart from the ones presented as use cases. With this tool, we enable an interactive exploration of parameter spaces and a better understanding of neural network models and grapple with the crucial problem of non-unique network solutions and trajectories. In addition, we observe a reduction in turn around times for the assessment of these models, due to interactive visualization while the simulation is computed.

## Introduction and related work

Neuronal models and neural mass models, usually based on coupled systems of differential equations, contain many degrees of freedom which determine the dynamics of the system. In a neural network, these models are interconnected and the strength of the interactions between elements can also change through time.

Since biological evidence to specify a complete set of parameters for a neural network model is often incomplete, conflicting, or measured to an insufficient level of certainty, parameter fitting is typically required to obtain outputs comparable to experimental results (see for example, López-Cuevas et al., 2015; Schuecker et al., 2015; Zaytsev et al., 2015; Schirner et al., 2016). And even *if* we had infinite experimental data available, Cubitt et al. (2012) have shown that, regardless of how much experimental data is acquired for a general system, the inverse problem of extracting dynamical equations from experimental data is intractable: “extracting dynamical equations from experimental data is NP hard.” This implies that in neural networks, the problem of finding the exact free parameters for a simulation leading to results matching experimental measurements cannot be solved in polynomial time, at least under the current understanding of computational complexity.

However, we can explore the parameter space with forward simulations in order to discover the system's characteristic behaviors and thus limit the search space to a computationally tractable sub-problem in an educated manner. The definition of these subspaces can then be the basis for robust—and non-arbitrary—parameter determination (in other words, mathematically valid performance function minimization). In fact, given the known mathematical characteristics of the dynamics of neuronal and neural mass networks, investigators should characterize the solution spaces of sufficiently complex networks and models before selecting what they propose are statistically diagnostic simulation trajectories. In practice, this rarely happens, even though parameter fitting without sufficient constraints and a rigorous exploration of the possible solution space can lead to conclusions valid only around local minima or around non-minima. Researchers frequently stay within arbitrary regions in the parameter space which show interesting behaviors, leaving other regions unexplored.

Visual parameter space exploration has been successfully applied in several key scientific areas, as detailed by Sedlmair et al. (2014). Combined with interactive simulation steering, the time for obtaining optimal parameter space solutions can be significantly reduced (Matković et al., 2008, 2014). Whitlock et al. (2011) present an integration of VisIt (Childs et al., 2005), a flexible end-user visualization system, into existing simulation codes. This approach enables *in situ* processing of large datasets while adding visual analysis capabilities at simulation runtime. A similar approach has been suggested by Fabian et al. (2011) for ParaView (Henderson, 2004).

Coordinated multiple views (CMVs) as proposed by North and Shneiderman (1997) and Wang Baldonado et al. (2000) can assist in visual parameter space exploration. CMVs are a category of visualization systems that use two or more distinct views to support the investigation of a single conceptual entity. For example, a CMV system can display a 3D rendering of a building (the conceptual entity) alongside a top-down view of its schematics—whenever a room is selected within the schematic overview, the 3D rendering will highlight the room's location. Roberts (2007) shows that CMVs support exploratory data analysis by offering interaction with representations of the same data while emphasizing different details. Ryu et al. (2003) present CMV systems that have been successfully utilized to uncover complex relationships by enabling users to relate different data modalities and scales, and assisting researchers in context switches, comparative tasks, and supplementary analysis techniques. Additional examples of such systems are presented by North and Shneiderman (2000), Boukhelifa and Rodgers (2003), and Weaver (2004).

Visual exploration of neural network connectivity, e.g., by displaying spatial connectivity data in 3D renderings, has previously been employed by scientists to better understand and validate models as well as to support theories regarding the networks' topological organization (Migliore et al., 2014; Roy et al., 2014). The infinite solution space of suitable connectivity paths and end configurations for neural networks makes fully automatic parameter fitting “hard,” since it involves satisfying multiple contradictory objectives and qualitative assessment of complex data, as explained by Sedlmair et al. (2014). Kammara et al. (2016) conclude that for multi-objective optimization problems, visualization of the optimization space and trajectories permits more efficient and transparent human supervision of optimization process properties, e.g., diversity and neighborhood relations of solution qualities. They also point their work toward interactive exploration of complex spaces which allows expert knowledge and intuition to quickly explore suitable locations in the parameter space.

To address efficient but rigorous parameter space exploration, we have developed an interactive tool for visualizing and steering parameters in neural network simulation models. In this work, we focus particularly on the generation of connectivity, since finding suitable connectivity configurations for neural network models constitutes a complex parameter search scenario. The generation of local connectivity is achieved using structural plasticity in NEST (Bos et al., 2015) following simple homeostatic rules described in Butz and van Ooyen (2013). We specify the problem from the control theory perspective, as variations in the structure system control the transition in its dynamics from an initial to a final state following a defined trajectory. The tool allows researchers to steer the parameters of the structural plasticity during the simulation, thus quickly growing networks composed of multiple populations with individually targeted mean activities. The flexibility of the software allows the exploration of other connectivity and neuron variables apart from those presented as use cases. We use CMVs to interactively plot firing rates and connectivity properties of populations while the simulation is performed. Moreover, simulation steering is realized by providing interactive capabilities to influence simulation parameters on the fly.

We have developed this tool based on two use cases where visual exploration is key for obtaining insights into non-unique dynamics and solutions. The first use case focuses on the generation of connectivity in a simple two population network. Here we show how the generation of connectivity to a desired level of average activity in the network can be achieved by taking multiple trajectories with different biological significance. The second use case is inspired by a whole brain simulation described in Deco et al. (2013), where the exploration of non-unique connectivity solutions is desired to understand the behavior of the model.

Applying this approach, an intractable inverse problem can be reduced to a tractable subspace, and the requirements for statistically valid analyses can be determined. Visualization can simplify a complex parameter search scenario, helping in the development of mathematically robust descriptions amenable to further automated investigation of characteristic solution ensembles. Observing the evolution of connectivity, especially in cases where several biologically meaningful paths may lead to the same solutions, can be useful for a better understanding of development, learning and brain repair. This work is a first step toward developing new analytic and computational solutions to specific inverse problems in neuronal and neural mass networks. Our software platform promotes rigorous analysis of complex network models and supports well-informed selection of parameters for simulation.

This paper is structured as follows: first, we present an introduction to generic dynamic neural network models from a control theory perspective. Next, we describe connectivity construction and its effects on the dynamics of the system. Then, the development process and design of the steering and visualization tool is detailed. The fifth section describes the results of using the steering tool in two different use cases. Finally, we discuss our results and present open questions and future work.

## General form of network dynamics

Let a neural network be defined by a set of ordinary differential equations in which *x*_1_(*t*), *x*_2_(*t*)…*x*_*n*_(*t*) are state variables of the system at time *t*. We assume that neurons in this model can be either in an active or quiescent state. The master equation of a neural network has been derived and explained in Cowan (1991) and Ohira and Cowan (1993). This equation provides a mathematical description of the evolution of stochastic neural networks in the form of a Liouvillian:

(1) $$\begin{array}{l} L=\alpha \overset{N}{\underset{i\;=\;1}{\sum }}\left(\Delta_{+i}-1\right)\Delta_{-i}+\overset{N}{\underset{i\;=\;1}{\sum }}\left(\Delta_{-i}-1\right)\Delta_{+i}\phi \left(\frac{1}{n_{i}}\overset{N}{\underset{j\;=\;1}{\sum }}\omega_{ij}x_{j}\right) \end{array}$$

where α is the decay function after a neuron has spiked, Δ_+*i*_ and Δ_−*i*_ are the raising and lowering operators which take a neuron *i* to and from an activation state, *n*_*i*_ is the number of connections to neuron *i*, *N* is the total number of neurons in the network, ϕ is the activation rate function which depends on the neuron model and ω_*ij*_ is the strength of the connection between neuron *i* and *j*. Synaptic growth and connectivity variations in neural networks further increase the complexity of the system. In the case of variable connectivity, the network master equation is transformed into:

(2) $$\begin{array}{l} L=\alpha \sum_{i\;=\;1}^{N}\left(\Delta_{+i}-1\right)\Delta_{-i}\\ \;+\sum_{i\;=\;1}^{N}\left(\Delta_{-i}-1\right)\Delta_{+i}\phi \left(\frac{1}{n_{i}(u(t))}\sum_{j\;=\;1}^{N}\omega_{ij}(u(t))x_{j}\right) \end{array}$$

where both ω_*ij*_ and *n*_*i*_ depend on the control signal *u* coming from the synaptic and structural plasticity algorithms at time *t*. We introduce this formulation to expose variables *u*(*t*) in the system, which can be controlled. We are interested in modifying these signals in order to induce changes in the network and thus achieve a target dynamic profile. However, it is worth noting that our approach is also applicable to non-stochastic neural networks.

### Control theory for network state trajectories

Both synaptic and structural plasticity can be seen as biological controllers in a multi-objective optimization problem. Under this view, the system gradually creates and destroys connections between neurons, or modifies the strength of existing synapses (control), to achieve a transition from one initial state to a final steady (or even homeostatic) state. This final state can be a previously known activity state which has been altered, as in repair after a lesion, or a new activity state to be achieved, as is the case in learning. Thus, the evolving connectivity problem can be mathematically expressed in terms of control theory as defined in Kirk (2012).

In our case, the control signals refer to the variations in the connectivity of the network while the states refer to the dynamics of the network. The state equations take the form of:

(3) $$\begin{array}{l} \dot{x}=a\left(x(t),u(t),t\right) \end{array}$$

where **u** is the history of control signals during the interval [*t*_0_, *t*_*f*_], and the state trajectory denoted by **x** is the history of state values during the same time interval. A control history which satisfies the constraints of the system (in this case, experimental parameters of neurons and synapses) during the time interval of interest is called an “admissible control.” On the other hand, an “admissible trajectory” is a state trajectory which satisfies the constraints of the state variables through the whole period of interest. The final state of the system is then required to lie in a specific region, defined as the target set, of the *n*+1-dimensional state-time space.

By applying the control signal **u**(*t*) from *t*_0_ to *t*_*f*_, the system will evolve from its initial state *x*_0_ following some trajectory to a final state *x*_*f*_. The “performance” of this trajectory is the difference between a desired and the obtained measure for a heuristic involving the dynamics of the system. In our case, the performance function is given by the homeostatic rules the system must follow. To reach a defined target activity regime, we cannot know *a priori* whether an optimal admissible control exists, which leads the system through an admissible trajectory for a given performance function. It may be impossible to find such a control history, and even if it exists, it may not be unique or numerically stable.

The optimization problem posed seeks a global minimum for one or more admissible trajectories of the system. For the class of neural networks described by the dynamical equations above, the problem of finding the exact control signals or free parameters for a simulation leading to experimental results cannot be solved in polynomial time. However, it may still be possible to confirm solutions in polynomial time.

## Connectivity generation in neural networks

Previous research by Sporns et al. (2005) has found that the assembly of anatomical connections among neurons, also known as the connectome, plays a fundamental role in explaining the high-level activities of the brain. However, the exact relationship between anatomical links and the functions performed by the brain has aspects that remains unclear. An attempt to model biologically realistic circuits immediately runs into the problem that the structure of the brain has yet to be comprehensively characterized. Existing connectomic datasets are incomplete or contain large uncertainties (Bakker et al., 2012). Conversely, information about the average electrical activity in specific brain regions is easier to acquire either directly, e.g., electroencephalogram, extracellular electrode recordings of spiking activity and local field potential, or indirectly, e.g., functional magnetic resonance imaging and optogenetics/calcium imaging.

Variations in the physical elements, which constitute a neural network, can be modeled using synaptic and structural plasticity. Structural plasticity, a model of the dynamic creation and deletion of synapses in a neural network, is desirable from two main perspectives. The primary purpose is to study the neurobiological phenomenon of morphological transformations that a neuron or set of neurons undergoes through time, leading to the creation or deletion of synapses. This phenomenon is part of brain development, learning and repair. However, a promising secondary role suggested by Diaz-Pier et al. (2016) is the automatic generation of neuron-to-neuron synapses to compensate for gaps in experimental connectivity data. Using structural plasticity, a network can autonomously generate synapses to achieve a stable desired profile of electrical activity, a measure that is experimentally more accessible than detailed connectivity data. By progressively and slowly changing the connections between neurons in the network and the weight of these connections for all regions, the structural plasticity algorithm is able to find stable configurations within the desired firing rate profile.

The structural plasticity implementation in NEST is based on the model proposed by Butz and van Ooyen (2013) and described in detail by Diaz-Pier et al. (2016). In this plasticity framework, neurons have contact points called synaptic elements which increase or decrease in number according to simple homeostatic rules. When new synaptic elements become available, they can be used to create new synapses. If the contact points are eliminated, the synapses formed earlier are destroyed. Homeostatic rules applied to the synaptic elements are intended to take the mean electrical activity to a desired state.

**Figure 1:**
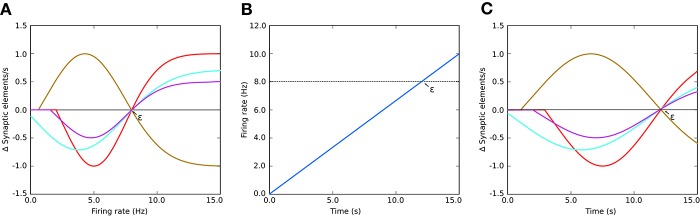
**(A)** Example of growth rate curves determining the rate of creation or deletion of synaptic elements in the structural plasticity model. The parameters which define the shape of the curve are two firing rates, the minimal firing rate for creating/deleting synaptic elements η and the target firing rate ϵ, and the growth rate ν which is the value of the curve in synaptic elements/s when the firing rate λ = (ϵ−η)/2. The red, cyan and purple curves have a negative value of ν which implies that synaptic elements will be deleted when the current firing rate is less than the target rate. These curves are therefore suitable for inhibitory synapses. Conversely, synaptic elements will be created when the current firing rate exceeds the target. The brown curve has a positive ν which works in the opposite way. All curves display different values of η; in particular, the cyan curve has a negative value of η. In these cases, all curves have a target firing rate ϵ of 8 Hz. It is important to note the slope of each curve close to the target firing rate ε; this slope is critical for the stability of the optimization algorithm. **(B)** Firing rate externally imposed on sample systems with Gaussian growth curves shown in **(A,C)** the resulting evolution of synaptic growth rate through time due to the firing rate changes depicted in **(B)**. See Figure 5B for an equivalent Gaussian growth curve for the two-population example in this paper, and the resulting free (not driven) dynamics in Figures 5C–E.

A Gaussian curve (Figure 1) is an example of a homeostatic rule describing the growth rate of connection points for neurons. The original model by Butz and van Ooyen (2013) uses intracellular calcium concentration as a proxy for the mean firing rate. In this paper's examples, we will use a variation directly referencing the mean firing rate as our homeostatic rule.

The parameters defining the growth and decay of synapses are the minimum firing rate η required to generate synaptic elements (or destroy them, depending on sign of ν), the value ν of the growth rate curve when the firing rate is (ε−η)/2, and the target firing rate ε. Modifying these values alters the way connectivity is created and destroyed in the network.

Thus, to calculate the number of synaptic elements per second (d*n*/d*t*) to create (or remove, if negative), we use:

(4) $$\begin{array}{l} \frac{\text{d}n}{\text{d}t}=vH\left[\lambda -\eta \right]\left[2{\mathrm{pow}}_{2}\left(-{\left[2\frac{\lambda -\eta }{\epsilon -\eta }-1\right]}^{2}\right)-1\right] \end{array}$$

where pow_2_*x* is the power function 2^*x*^ and H[*x*] is the Heaviside step function equal to 0 when *x* < 0, otherwise 1. Equation (4) is equivalent to the Gaussian used in Diaz-Pier et al. (2016) after directly replacing the calcium concentration with the firing rate λ. In this paper's simulations, this form is not biologically motivated, but is a homeostatic meta-rule being used to numerically solve for networks consistent with fixed firing rates.

The firing rate λ at time *t* used in Equation (4) is calculated by low-pass filtering spike train data by convolving that data with an exponential decay kernel (Park et al., 2013): the current firing rate λ is increased by 1/τspikes/s for each spike and decays exponentially with a time constant τ = 10s between firing times. Thus,

(5) $$\begin{array}{l} \tau \frac{\text{d}\lambda }{\text{d}t}=-\lambda +\underset{t^{f}}{\sum }\delta \left(t-t^{f}\right) \end{array}$$

where *t*^*f*^ are the firing times of the neuron and δ is the Dirac delta function. This calculation is internal to NEST and independent of our tool. When the convolution technique isn't suitable, an alternate mean firing rate can be computed using a user-defined window size applied to binned spike trains.

As discussed in the previous section, synaptic and structural connectivity can be seen as multi-objective optimization algorithms which take the network from an initial state to a final state where *something has been learned* or *a new activity pattern has been enabled*. Partial information about the connectivity can be combined with information about average activity in the system to initialize models of structural plasticity filling the gaps in the constraints of the system. However, finding suitable connectivity configurations and generation trajectories for neural network models is non-trivial, which is exacerbated by the nature of experimental data. The known experimental data often fails to sufficiently constrain the model to parameter subspaces that can be completely explored with reasonable resources within reasonable time frames.

Enabling structural plasticity for a single population to reach a targeted activity level is usually unproblematic, fast, and relatively insensitive to the choice of parameters such as ν and η. However, a big challenge arises when structural plasticity is involved simultaneously on several interconnected populations with differing levels of activity. Even small changes in the connectivity of each population will impact the activity of all others to which it is connected, leading to a propagated destabilization. Another parameter which has a great impact on stability is the update interval at which synapses can be deleted or created. As in any control system, the delay between a control change and the response of the system strongly determines the capability of the controller to keep the system in a stable region.

In Diaz-Pier et al. (2016), the simulations were performed statically, meaning no steering was possible during runtime. Due to the large combination of parameters to be controlled and variables to be observed during the search process, brute-force parameter search based on static simulation proved to be insufficient to obtain stable states. The selection of adequate parameters to define and constrain the growth of network connectivity, especially for multi-population or coupled networks, is not trivial since some values might lead to unstable setups. Therefore, modifying the characteristics of the growth behavior (ν and η see Figure 1) for each population and the update interval *during simulation* becomes crucial for finding a suitable stable state for multi-population networks. We use the terms “population” and “region” interchangeably to refer to groups of neurons. The term chosen depends on the use case. In general, a region contains one or more populations while populations specify groups of neurons of the same type. Connectivity exists both within and between populations and regions. All types of connectivity can be subject to plasticity or remain fixed after setup. The software can be modified to take into account any number of populations per region, arbitrary types of neurons, and any number of regions. The user can also specify different types of connections between the same populations and apply various structural plasticity rules to each of them. The user can choose between a variety of connectivity modalities in NEST, ranging from one-to-one, all-to-all, fixed in-degree, fixed out-degree, fixed total number of connections, and pairwise Bernoulli. However, structural plasticity support is only currently implemented for one-to-one and all-to-all connectivity. Other modalities can be used, but structural plasticity will not affect these connections.

In the context of a simulation with evolving connectivity, the dynamic nature of the parameter search workflow derived from the two use cases presented later requires:

W1: The simultaneous analysis of several changing variables by an expert.
W2: Comparing the level of activity of several populations simultaneously.
W3: Changing simulation parameters at any moment in each population of the network.
W4: Snapshotting a time point in the simulation and storing the connectivity state.
W5: Loading a previously stored connectivity state.

This workflow can potentially be assisted with an interactive tool enabling scientists to explore and steer such simulations within the space of possible trajectories. To achieve this goal, a scientist needs interactive feedback on the number of connections and the level of electrical activity in all populations.

## *In situ* visualization and steering of connectivity generation

To enable navigation through the connectivity generation parameter space, we developed a tool enabling interactive steering and visualization. The development was driven by the need to rapidly reach stable configurations of connectivity in multiple tightly connected populations. We then extended the tool to support further use cases which are presented later. The tool allows for the visualization of trajectories that the system undergoes during simulation by showing the changes in the observable states of the network (specifically the activity and connection properties of the network). In addition, this tool allows for the modification of the control signals for the generation of connectivity, i.e., the plasticity algorithm's parameters.

The developed tool realizes a CMV system by applying principles of event-driven architectures as presented in Abram and Treinish (1995), Michelson (2006), and Nowke et al. (2015). The development of the tool was organized into four stages: first, the simulation script was modified to retrieve electrical activity and connectivity values; second, the visualization components and user interfaces were developed; third, processing of parameter changes from the user interface was added; and finally, the simulation script was optimized to run on supercomputers.

In the first step, we started by reproducing the plots from the non-interactive analysis workflow used in the second use case. This initial design phase revealed the following visualization requirements (**R1–R5**), followed by the requirements for simulation steering (**R6–R10**). These requirements hold for all presented use cases:

R1: Deal with at least 2 × *N* representations of time series data (electrical activity and connectivity), where N is the number of populations in the simulation.
R2: Interactively plot the firing rate for selected populations. The firing rate from the last simulation step should be displayed as soon as its computation concludes.
R3: Interactively plot connections for each population. As for the firing rate, the latest total connections per population should be displayed.
R4: Enable the selection and filtering of populations for plotting and further investigation. The means to select and filter populations of interest must be provided.
R5: Have a well defined way to distinguish populations in the plot. Since multiple populations can be selected for comparison, visual clutter needs to be avoided.
R6: The user interface must allow for the modification of each population's growth rate ν and apply each value in the simulation.
R7: The user interface must allow for the modification of a population's minimum electrical activity η and transfer the new value to the simulation engine.
R8: The user interface must allow for the modification of the update interval and transfer its change to the simulator.
R9: Control the NEST simulation from within a graphical user interface. Provide the means to start or stop the simulation, trigger the saving and loading of a network state, and allow convenient access to the visualizations.
R10: Enable loading and saving of the current network state (connections and user controlled parameters).

**Figure 2:**
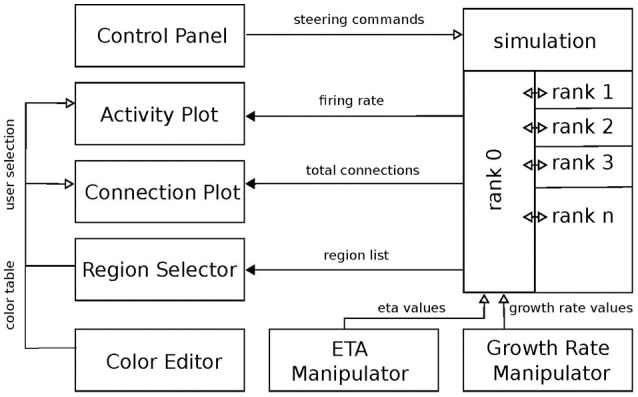
Overview of the system architecture: boxes denote individual services. Black arrows mark communication from the simulation engine to the visualization front-ends. Vice versa, white arrows indicate event-flow from the visualization services to the simulation engine. Ranks indicate individual MPI processes responsible for the parallel computation of the neural network. The “ETA” (η) and “growth rate” (ν) manipulators control the respective variables from Figure 1.

Requirements **R1–R5** cover the parameter search workflow **W1** and **W2**. **R6–R10** target **W3–W5**. Based on these requirements, we developed the software architecture as depicted in Figure 2. Each box in this figure we term a service. Services and the simulation engine NEST exclusively communicate via events. Communication via events allows us to treat each visualization as an independent loosely-coupled service. One benefit of this approach is that all services are independent of each other, facilitating the production of small reusable software components that are easy to maintain and can be reused in different contexts.

Event-communication is realized with the “*nett* ” messaging framework (see Supplementary Material), which is an open source C++ network library facilitating data transfer between application boundaries based on the publish and subscribe pattern. To enable communication between applications, *nett* provides *slots*. A slot is an unidirectional communication channel strictly typed to an event. Slots exist in two flavors: out-slots for publishing events and in-slots for subscribing to these. Consequently, subscribing slots can be connected to several publishers emitting the same event. An event is defined via a customizable schema, describing the fundamental data types the event is composed of. Moreover, *nett* provides Python bindings, making it possible to communicate between Python, i.e., the visualization implementations, and C++ applications, i.e., NEST.

Streaming simulation results from NEST is already possible with the MUSIC interface (Djurfeldt et al., 2010). However, MUSIC is specifically built for transferring large arrays of structured data in parallel with a certain step size and with a focus on latency. It is tailored to multi-scale coupling and large data transfer. In comparison, *nett* focuses on arbitrary serialization of data through tiny pipes and is based on a publish-and-subscribe communication mechanism. In addition, it is intended for point-to-point continuous streaming and is event-driven in comparison to the pull-driven communication regime by MUSIC. Furthermore, *nett* offers routing discovery while MUSIC relies an a static configuration on startup. In summary, *nett* is tailored to concise and light data transport and easy to integrate data streaming from C++ or Python codes.

The rest of this section will outline the required simulation instrumentation and the visualization services in more detail.

### Simulation instrumentation

Interactive steering relies on a bidirectional communication between the visualization and steering interfaces to a simulator. In our setup, activity levels and connectivity from populations computed by NEST are transferred via event communication over a network connection to the visualizations, where users can modify parameters of the simulation model, which in turn are fed back to the simulator. The values of interest are the firing rate of each population which serves as a proxy for electrical activity and a population's total connections formed due to connectivity generation. These are the observable states of the network. Steering parameters are the minimum firing rate η and the growth rate ν of each population, the update interval for the connectivity generation, and finally, basic commands to NEST such as ending or resetting the simulation, and storing or loading the current network state.

To retrieve firing rates and total connections, instrumentation of the simulation script is required. To this end, the simulation acquires the latest firing rates and total connections of each population in each iteration and publishes these as events. Then, parameter changes from the graphical steering interfaces, asynchronously retrieved during the model's computation, are applied and the next iteration is continued.

To adapt a NEST simulation to a different use case, the first step consists of determining what data needs to be transferred from or to the simulation. The next step consists of creating an event definition schema for the data to be transferred if one is not yet present. Then, slots for communicating this data definition can be created: out-slots for publishing data and in-slots to retrieve it. Once slots are created, in-slots need to be connected to their corresponding out-slots. Any in-slot should be used in a thread to asynchronously retrieve data without blocking the computation of the simulation. Once an event is received by a slot, its data needs to be applied in the next iteration of the simulation. In a complementary fashion, out-slots send the simulation results for each iteration by retrieving values of interest from the simulation and filling the slot's event and sending it. The same methodology is used for visualizations or graphical user interfaces which are use case specific.

### Visualization system overview

The visualization system consists of six services fulfilling the above listed requirements. A demonstration video of the tool can be found in Supplementary Material (see video Movie 1). In the following, we outline each service and its responsibility in the workflow.

#### Control panel

**Figure 3:**
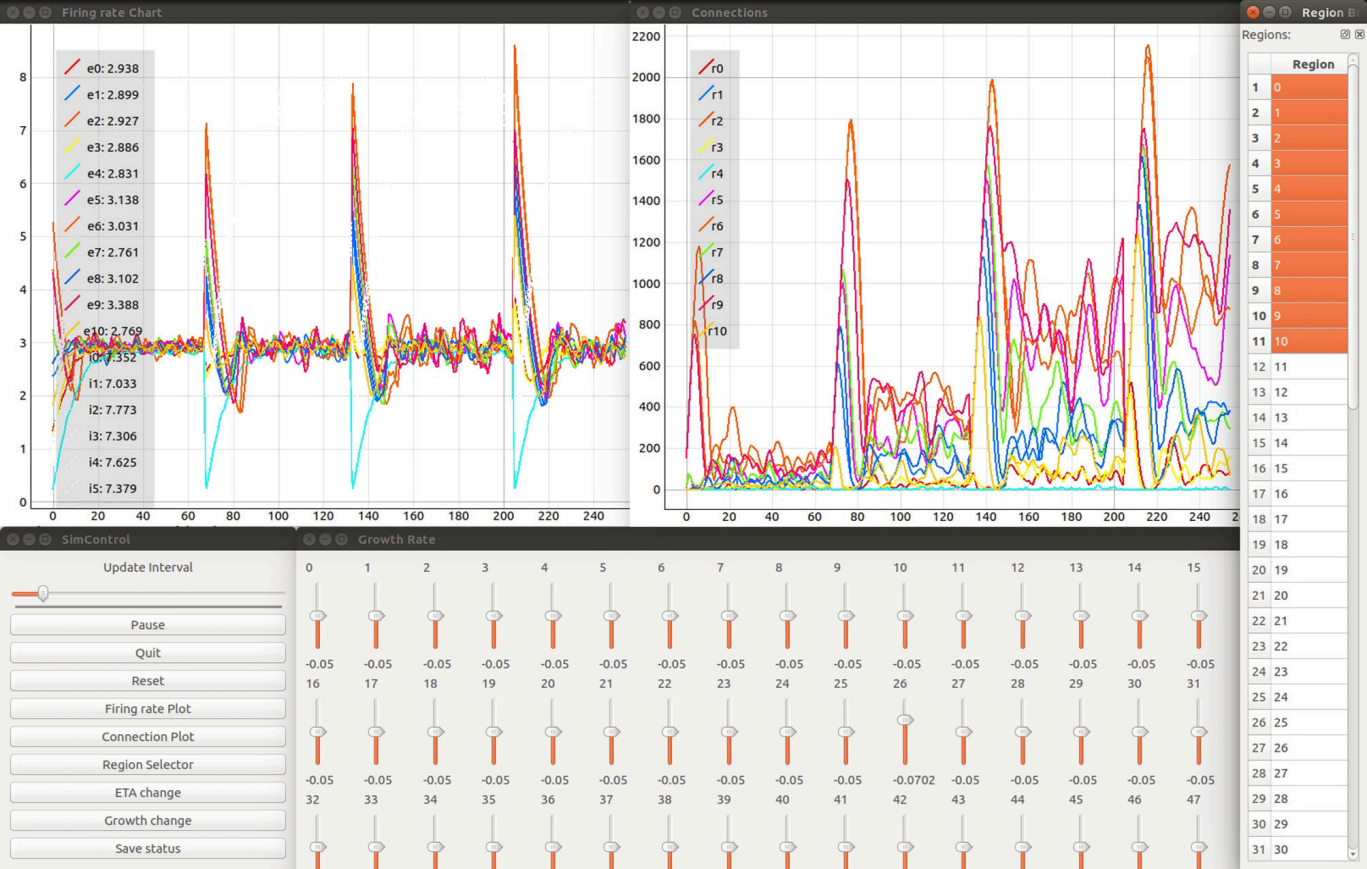
Firing rate in spikes/s of simulated brain regions **(Upper left)** and total connections **(Upper right)** are retrieved while a NEST simulation is performed. Time is measured in number update intervals. The steering interfaces (*Control Panel* and growth rate manipulation; bottom left and center) allow interactive parameter space exploration which is synchronized with the current simulation. The growth rate (in Δ synaptic elements/ms) for each region can be controlled using the corresponding slider. The region selector (far right) provides the means to filter the brain regions of interest depicted in the plots. The legends provided in each plot denote the current selection from the region selector along with the color used to identify the corresponding curve. Specifically in the example shown, the labels *e0*- *e10* and *i0*- *i5* identify the average firing rate for excitatory and inhibitory populations in network regions 0-10 accordingly. Labels *r0*- *r10* identify total outgoing connections from network regions 0–10. Please refer to section 5.2 for more details on the network model used in this example. Please refer to the video Movie 1 in Supplementary Material, for a detailed explanation of the tool's interface.

The *Control Panel* is the central place to provide convenience functionality, i.e., to start the simulation, all visualization services, steering interfaces, the *Color Editor*, and *Region Selector* (see Figure 3). It serves as an entry point for users to start the investigation of structural plasticity. The user interface facilitates changing the update interval (**R8**) and allows the simulation to be paused or restarted (**R9**). In addition, it provides a graphical interface for loading and saving the network state (**R10**).

#### Region selector

The *Region Selector* is a graphical interface displaying a list of all populations in the simulation (see Figure 3, rightmost element). These populations are defined by the network modeler in the simulation script as part of the instrumentation process. This is detailed in the instrumentation manual in Supplementary Material. The list provides the means to select populations of interest whose connectivity and firing rates should be plotted (**R4**). To this end, the *Region Selector* retrieves the number of populations from the simulation (see Figure 2). The user can then select multiple populations by clicking on them. All connected visualizations are linked with the current selections; thus it can be used to synchronize all tools for filtering data and in this way populations of interest can be focused (**R4**). The *Region Selector* can also be used to inspect individual populations of interest. By double clicking on a population in the list, an additional *Activity Plot* and *Connectivity Plot* is created plotting only the selected population of interest. This functionality can be used on multiple populations, independently of selections performed later on and facilitates the pairwise comparison of populations.

#### Activity plot

The assessment of the simulation results is based on the inspection of a population's firing rate. The *Activity Plot* is an interactive service that plots the firing rates of populations selected in the region selector (**R1**). It is used to visualize the trajectories that the network traverses in terms of its functional states. To this end, it connects to the region selector and listens for incoming selection events (**R4**). To display the firing rate (**R2**), the service directly connects to the simulation to retrieve the last iteration result. Interactive zooming and panning capabilities allow the scientist to focus on details on demand, following the “information seeking” mantra postulated by Shneiderman (1996). Interactive zooming can be used to zoom into a specific time interval and assess the depicted curve in more detail. Panning allows the user to move the selected time interval of interest, effectively moving the curve to the left or right. The information seeking mantra states that users should be able to get an overview first, then zoom and filter the data, and finally query details on demand. Furthermore, axes can be independently scaled or their data range confined. In the *Activity Plot's* initial configuration, which can be modified by the user, both axes will be scaled in such a way that all retrieved firing rate values are visible. The tool also allows the user to export the plot as a figure. To distinguish multiple curves, a color table can be defined via the *Color Editor* (**R5**), as discussed below. A legend in the upper left corner relates the selected populations to the depicted curves shown in Figure 3 in the upper left window. In addition, it shows the latest firing rate next to each population's legend label. The legends can be changed by the user of the tool. In this work we use the label *e* and *i* to identify excitatory and inhibitory populations and a number to identify the region they belong to. Individual *Activity Plots* can be used in conjunction with the region selector by specifying a population of interest. Therefore, multiple plots can be used for comparison tasks (**R1**). In this setup, the visualization ignores user input and is fixed to the initial selection.

#### Connectivity plot

The *Connectivity Plot* (see Figure 3, upper right window) displays the total number of connections for a population in accordance with **R3**. Since structural plasticity is responsible for a change in the total connections depending on the population's firing rate, the plot is the primary means to verify the structural plasticity model. It shows the trajectories of the network in terms of its structure. This visualization is connected to the region selector and thus enables filtering of the populations to be displayed (**R4**). Analogously to the *Activity Plot*, it is linked to the *Color Editor*. Whenever attributes like color, line-style-drawing, or thickness are changed, these values are applied. The legends can be changed by the user of the tool. In this work we use the label *r* and a number to identify the total connectivity values for an specific region. Like the *Activity Plot* service, it offers interactive zooming and panning functionality. Likewise, axes are automatically scaled such that all retrieved connectivity values are depicted. In addition, plots can be exported as figures for publication purposes or the tracking of results.

#### Color editor

The *Color Editor* provides a graphical user interface that mediates the customization of color, line drawing style, and line thickness for each population's firing rate and connectivity plots. Whenever the user changes an entry, a “color changed” event is emitted and processed by the *Activity Plots* and *Connectivity Plots*. In addition, the color table is saved to disk for later reuse. Its primary use is to help in distinguishing curves within the plotting visualizations (**R5**). The *Color Editor* enables the customization of the depicted firing rate and connectivity curves in the plot. Here, users can select a color for a population's inhibitory (I) and excitatory (E) population by clicking on the corresponding list entry. In addition, line drawing style and thickness can be controlled. The population's name is equal to its specified counterpart in the simulation.

#### Manipulation of structural plasticity parameters

The user interfaces for η and ν are the primary means of steering the simulation for the parameter space exploration (**R6** and **R7**). This interface allows for the modification of the control signals, enabling the structural plasticity algorithm to take the system from its current state to a desired final state (see Figure 3, bottom center). Both steering interfaces are designed as separate standalone services that can be started within the *Control Panel*. The η and ν services provide graphical user interfaces, each presenting one slider for each population. Their influence on the creation or deletion of synapses is indicated in Figure 1. Each slider is named according to the population's label and shows the current value used in the simulation. Whenever the user changes a value by adjusting the slider, an event is emitted which is subsequently processed and applied by the simulation in its next iteration step. The upper and lower limits for the control parameters can be defined inside the scripts for each controller interface. Please refer to the instrumentation manual in Supplementary Material, for more details.

#### Loading and saving network states

To re-use previously found connectivity patterns in neighboring points of the parameter space, we implemented a save and load functionality (**R10**). The current values for η and ν are saved for each population as well as the connectivity update interval. All current connections between all neurons are also saved. These connections are defined by a source neuron, a target neuron and the synapse model which links them. Finally, the total number of connections for each population are exported to a file which can be used in the next phase of the simulation loop.

To re-use a previously created snapshot, we first load the types of all synaptic elements for each population. When using the structural plasticity framework in NEST, the first step consists of defining the plastic synapses. This requires the specification of a synapse model as well as the definition of pre- and post-synaptic elements between which a synapse can be created. The growth curves for these synaptic elements are reconstructed using the stored values for η and ν. Then the synaptic elements are registered in the structural plasticity framework and the update interval is set for the simulation. This is performed by using the set status functions of NEST through PyNEST/CyNEST (Eppler et al., 2009; Zaytsev and Morrison, 2014). Finally, all connections are recreated, marking them as non-static links which can be modified by the structural plasticity algorithm. In this way, a new network with differing global parameters such as global coupling or inhibitory strength can start from a partial solution and arrive at the target activity values more quickly. For more details about the implementation of the structural plasticity framework please refer to Diaz-Pier et al. (2016). This functionality can be triggered from the *Control Panel*.

## Results

**Figure 4:**
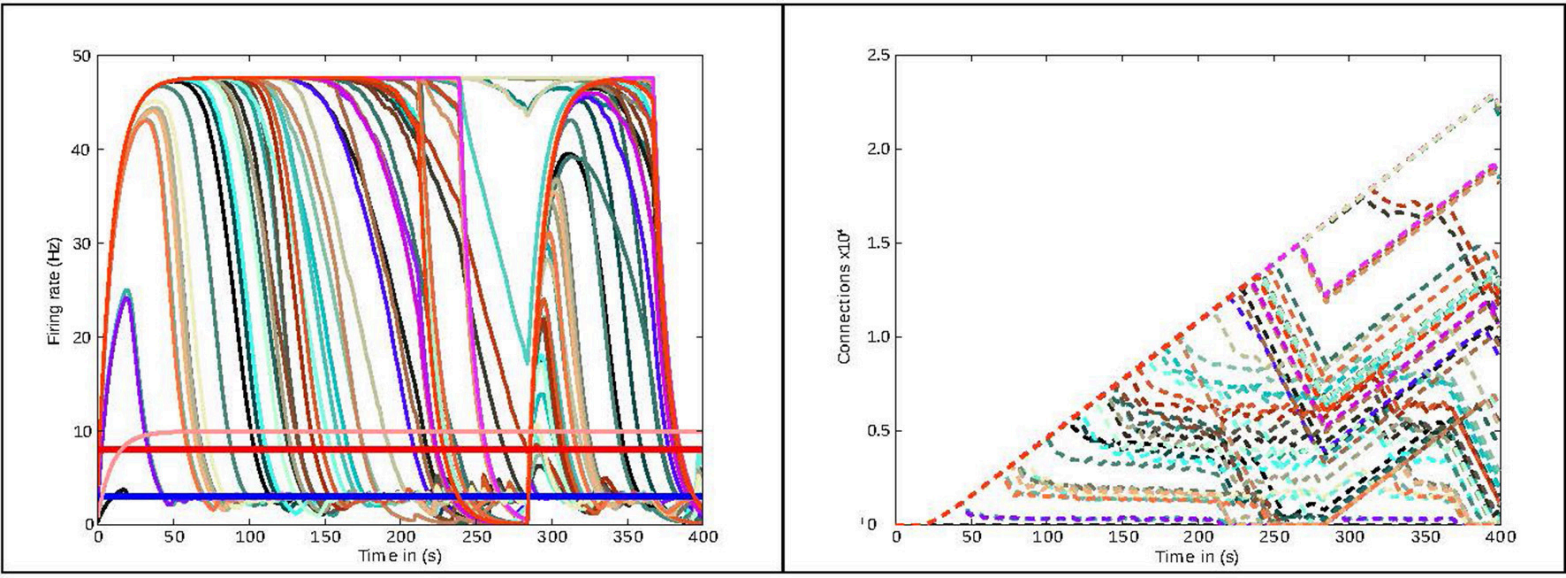
Previous method of visualizing simulations: visualization of the simulation as performed before the presented tool was developed. The figure shows the evolution of the average firing rate for each region (solid curves) and numbers of outgoing connections (dashed curves) from each region using structural plasticity in a non-interactive (static) experiment. Each color represents a different population. In this static approach, a large number of independent simulator runs are performed over a predetermined, non-interactive parameter space and then displayed with *ad hoc* scripts. Mapping the non-physiological solutions with saturated firing rates onto regions of the parameter spaces is highly non-trivial (compare approach with Figure 3).

In this section, we present the results obtained from two use cases in connectivity generation. For the first use case, the results of running structural plasticity simulations before the interactive visualization tool was developed were previously reported in Diaz-Pier et al.(2016, Figure 5, section 3.3.1). Figure 4 (from this current paper) shows the equivalent output for the second use case, reflecting the previous visualization approach. Due to the large number of unlabeled curves, the inability to focus on data for particular populations and the lack of interactivity with the visualization, using this static approach makes it very difficult for the user to identify the evolution of connectivity in relation to parameter changes. Moreover, a new simulation run is required whenever any parameter needs to be changed. Even when some regions have easily reached the target activity of 3spikes/s, for some set-ups it is extremely challenging to identify suitable trajectories that lead to stable solutions for all populations.

**Figure 5:**
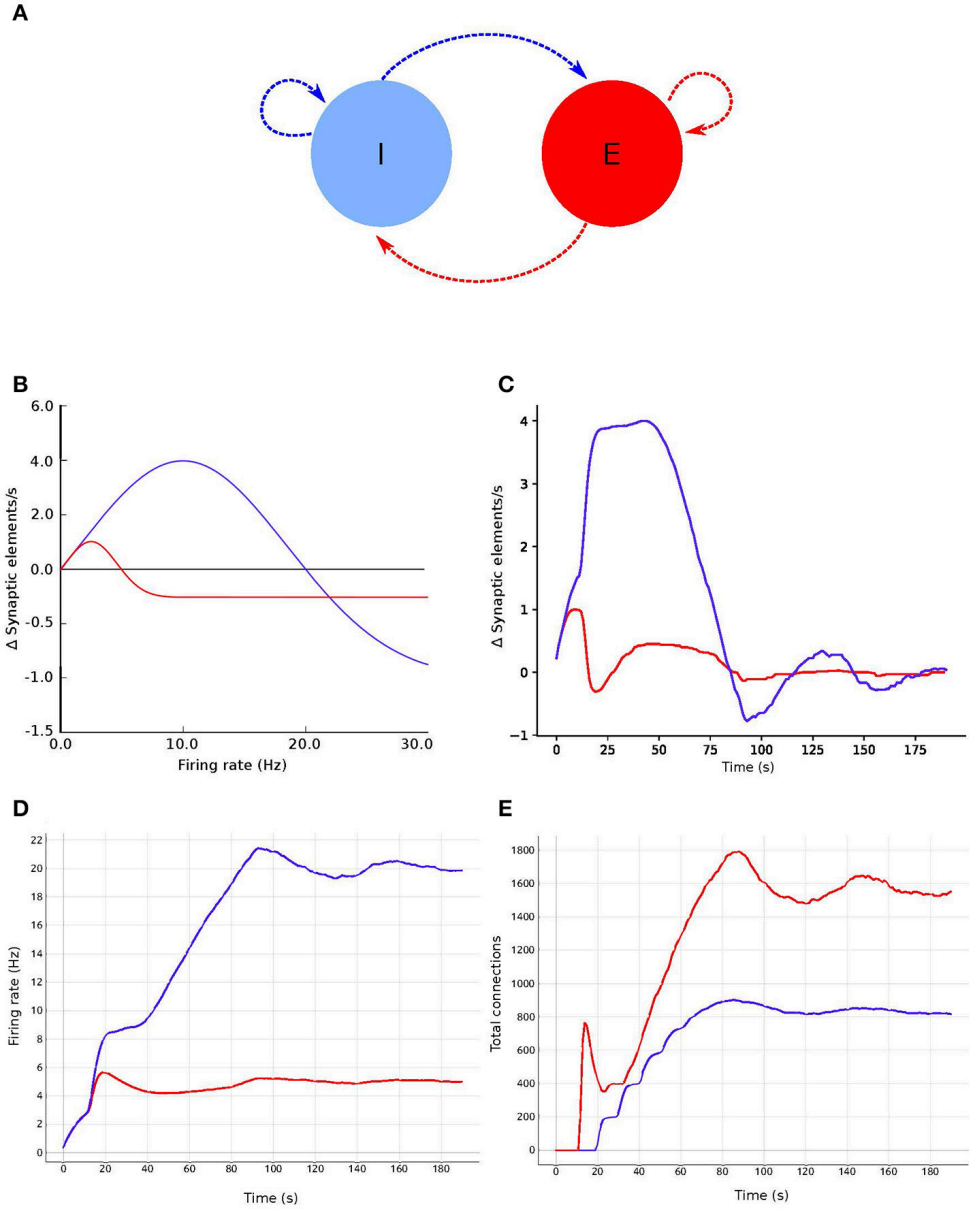
Evolution of firing rate and connectivity for the two population example: **(A)** abstract view of the model consisting of two populations, one excitatory (red) and one inhibitory (blue) with respectively excitatory connections (red arrows) and inhibitory connections (blue arrows), both controlled by structural plasticity; **(B)** Gaussian growth curves mapping current firing rate to growth rates (see Figure 1); **(C)** growth rate dynamics; **(D)** evolution of the firing rate; and **(E)** evolution of the total number of connections during the simulation. Colors in **(B–E)** are as in **(A)**.

In this type of simulation, the system is constrained by connectivity data and desired activity levels obtained from experimental measurements. However, these constraints still allow the system to reach non-physiological states such as saturating at high firing rates (see Figure 4). Moreover, the system may follow several trajectories to reach these implausible states, indicating that the system is under-constrained. On the other hand, there are many admissible trajectories which take the system to biologically plausible states. Biologically meaningful trajectories should be identified by heuristics, expert knowledge, and further experimental measurements gained through a deeper understanding of the parameter space to which the neural circuit is subject. At first glance, it is not clear how to explore the parameter space in these complex systems, as the large number of variables and long simulation times make it unfeasible to find stable populations through a brute force approach, and no heuristic is available to reduce the dimensionality. Without expert knowledge in a closed loop setup, admissible trajectories are fundamentally hard to find.

In the following sections, we demonstrate the challenges of parameterizing network models and the potential for an interactive visualization and steering tool, such as the one we propose, to address them. All experiments have been implemented with NEST 2.10.0 (Bos et al., 2015) and its Python language bindings which are described in Eppler et al. (2009); Zaytsev and Morrison (2014). The complete NEST scripts used in this work can be found in a GitHub repository. For more details, please see Supplementary Material.

### Two population model

In this use case, we create a model with two populations of point neurons, one excitatory and one inhibitory as shown in Figure 5A. The whole network contains 1,000 leaky integrate-and-fire neurons with exponential-shaped post-synaptic currents, of which 80% belong to the excitatory population and the rest to the inhibitory population. Parameters for the point neurons are listed in Table 1. All neurons receive independent background excitatory Poisson noise at a rate of 10kHz. At the beginning of the simulation, no connections between neurons are present. The system is allowed to create both excitatory and inhibitory connections (red and blue dashed arrows, respectively, in Figure 5A), using the structural plasticity framework in NEST. The weights for the created synapses are 1 and −1 respectively. The evolution of the firing rate (Figure 5C) and the growth of connections (Figure 5C) is regulated by two homeostatic rules defined by Gaussian curves, as shown in Figure 5D. The target average activity of the inhibitory population is set to 20Hz while the target average activity in the excitatory population is set to 5Hz. Figure 5C shows the evolution of the growth rate for excitatory synaptic elements in both populations during a simulation. These dynamics originate from the fixed firing rate curves shown in Figure 5B. The structural plasticity algorithm uses that relation at every simulation step to decide how many connections to create or delete.

**Table 1:** Network parameters for the first and second use cases.

**Parameter**	**Value**
Capacitance of the membrane *C*_m_	0.25 nF
Resting potential *V*_L_	−65 mV
Threshold membrane potential *V*_thr_	−50 mV
Reset membrane potential *V*_res_	−65 mV
Refractory time τ_ref_	2 ms
Growth rate excitatory synaptic elements	0.0001 elements/ms
Growth rate inhibitory synaptic elements	0.0004 elements/ms

The evolution of the connectivity generation can be guided by modifying the growth rate and shape of the Gaussian curve linked to each type of connection. Figures 5D,E show an example of this process. In this use case, an interesting feature to observe using the visualization and steering tool is the path to the solution. With the configurations used here, one can see how allowing faster growth of inhibition triggers an overshoot in the generation of excitatory connection to compensate. As a result, a rewiring of the system is obtained. These paths to the solution can be linked to onsets of critical periods in learning and healing or by external stimulation (Hensch, 2005). By regulating the speed of the creation of connections in the system, scientists can explore different paths to solution where the relationship between excitation and inhibition changes through time.

**Figure 6:**
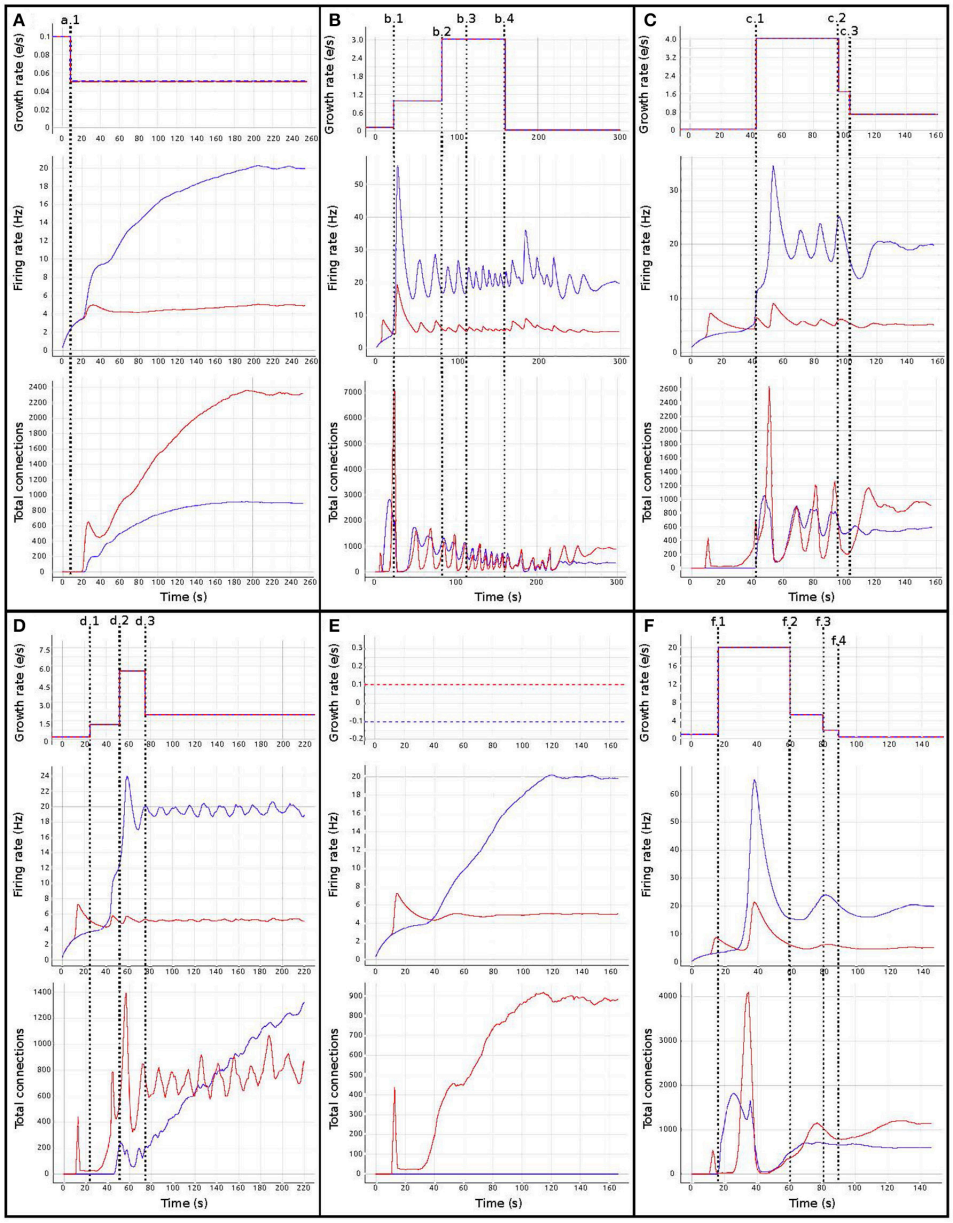
Evolution of growth rate (Top), firing rate (Middle), and outgoing connections (Bottom) for six different trajectories **(A–F)** in the two population model use case, excitatory (red) and inhibitory (blue). Vertical dashed lines correspond to manual changes using the graphic interface to the growth rate (top curves) or update interval (at b.3) control variables. All other simulation parameters are held constant for all runs, including initial growth rate. Please see the main text for a discussion of the features of each set of trajectories.

Figure 6 shows the evolution of growth rate (synaptic elements/s), firing rate (Hz) and connectivity (total number of connections) for six examples of the multiple trajectories and connectivity configurations that the network can show. All examples start with an initial growth rate of 0.0001 synaptic elements/ms. Figure 6A shows a smooth growth similar to Figure 5, but where the control signals have been modified to reduce the overshoot in the inhibitory population. That is done by reducing the initial growth rate to 0.00005 at iteration 8 (mark a.1). Figure 6B shows an example of a simulation where the control signals for growth start with aggressive growth values, producing a constant oscillatory behavior. That is achieved by changing the growth rate from 0.0001 to 0.0010 at iteration 38 (mark b.1) and then to 0.0030 at iteration 80 (mark b.2). Following these signals, the connectivity update interval is increased to 500 ms (from the standard length of 100 ms), which produces a big oscillation, triggering a rewiring of the network (mark b.3). Finally, growth is reduced to a slower pace, which helps the system settle at a stable state. This reduction is achieved by setting the growth rate to 0.00005 at update 161 (mark b.4). The final connectivity is very similar to the one reached in Figure 5. This example shows a different trajectory which reaches the same final state.

Figure 6C illustrates very fast initial growth by changing the growth to 0.004 at iteration 46 (mark c.1). Then, a sharp reduction in growth when the system oscillates near the target firing rate. The growth rate is changed to 0.0018 at iteration 98 and further down to 0.0007 at iteration 103 (marks c.2 and c.3 accordingly). Figure 6D shows a case which seems stable in terms of activity, but is unstable in terms of connectivity, as it exhibits a constant race between excitation and inhibition in the connectivity to maintain the target activity. The growth rate is set to 0.001 at iteration 24 (mark d.1), to 0.0056 at iteration 52 (mark d.2) and to 0.0020 at iteration 78 (mark d.3). Figure 6E shows a trajectory which is not biologically meaningful. This network has been built only from excitatory connections by modulating the growth of connections very carefully around the target activity. Here, we have defined a growth curve that does not allow the creation of inhibitory connections unless the activity is above the desired firing rate. Finally, in Figure 6F, we see a trajectory which is not admissible (not biologically meaningful) because the network is taken to an artificially high firing rates before it settles back to its target. At iteration 17, the growth rate is set to 0.002 (mark f.1) and then slowly reduced to 0.00056 at iteration 60, to 0.0002 at iteration 80 and finally to 0.00005 at iteration 90 (marks f.2, f.3, and f.4 accordingly). This graph shows how a network can traverse biologically inadmissible trajectories and still reach the target activity.

These results show that several instantiations of the same system using different dynamics lead to the same target activity but different connectivity patterns. Visualization and steering is fundamental for producing, observing, studying and cataloging these behaviors in the network. See Bahuguna et al. (2017) for an example of the same phenomenon exhibited in a more complex network. Thus using target activity as a tuning parameter without this kind of exploration leads to selecting one of these network connectivity states arbitrarily. The resulting model may not be representative of the kinds of networks that produce this activity, or of the target system to be modeled.

In other words, the target activity does not uniquely identify a network, or even a contiguous volume of parameter space, but is the property of a distribution of distinct networks distinguished by parameters that are not the direct targets of research—this class of inverse problem is degenerate. The network structure may be critically path-dependent, dependent upon parameters which are stochastic sequences (external control variables) or even dependent upon numerically unstable parameter functions. Simple networks such as the one shown in this example are frequently used in computational neuroscience but rarely with consideration to the careful characterization of the parameter spaces. Thus, in the absence of analytical methods to identify alternative solutions in the parameter space, steered visualization is a highly effective method for producing, observing, comparing and cataloging network configurations.

### Whole brain simulation

**Figure 7:**
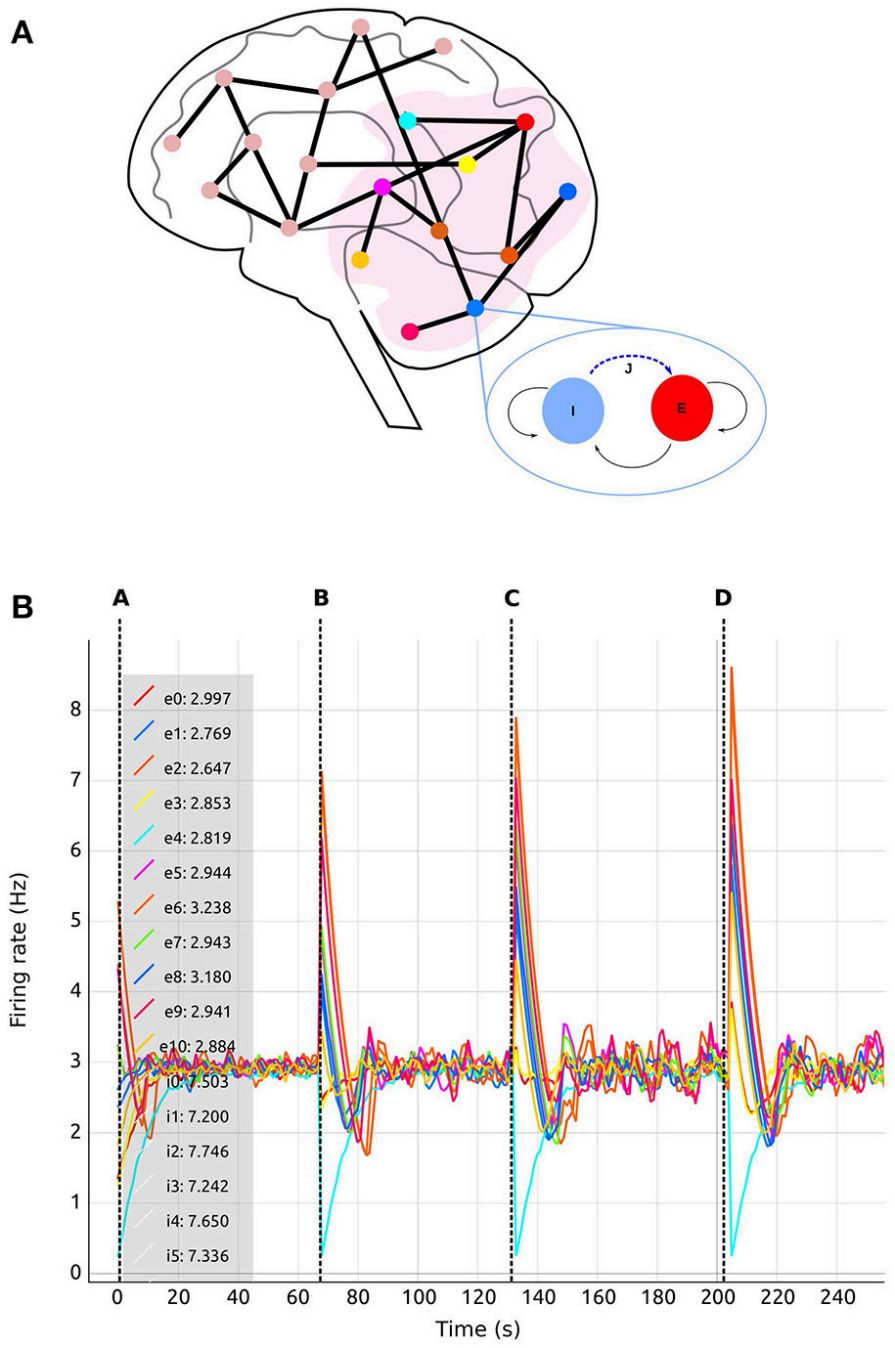
Use case 1 inspired by Deco et al. (2014) whole brain model. **(A)** Abstract representation of the whole brain model including 68 regions. A subset of the regions is selected (pink area). The zoom-in view of one of the regions shows the abstract model of each region, consisting of two populations, one excitatory (red) and one inhibitory (blue). Inhibitory connections to excitatory neurons in the same region (blue dashed arrow labeled *J*) are subject to structural plasticity. **(B)** *Activity Plot* (see section 4.2.3) of selected regions (0–10) as a function of biological time. Regions are numbered from 0 to 67. Tags *eX* and *iX* identify curves for excitatory and inhibitory populations in the *Xth* region. A legend (upper left) indicates the current selection. The number following the colon after the tag is the region's firing rate during the last simulation step. Vertical dashed lines separate sections of the simulation with differing values of the global connectivity coupling (see section 5), *G* = (A) 0.5; (B) 1.0; (C) 1.5; (D) 2.0. The vertical dashed lines are superimposed on this plot and are not part of the *Activity Plot* service. Increases to the global coupling parameter lead to an increase in the strength of the connections between regions. The firing rate spikes initially as a response to this change; in response, structural plasticity modifies connectivity according to the homeostatic rules until the firing rate stabilizes again closer to the target firing rate.

This use case is inspired by a previous study by Deco et al. (2014). The experiment consists of a whole brain simulation using 68 interconnected brain regions, each of which represented by a spiking network containing 200 conductance-based leaky integrate-and-fire neurons, as illustrated in Figure 7A. The original work by Deco et al. (2014) uses a Dynamic Mean Field Model (DMFM) originally developed in Wong and Wang (2006).

The coupled non-linear stochastic equations of the DMFM describe the behavior of mean-field neuronal regions and their influence on each other:

(6) $$\begin{array}{l} \;\dot{s}=s/\tau_{s}+(1-s)\gamma H(x)+\sigma \nu (t)\\ H(x)=(ax-b)/(1-\exp(-d(ax-b)))\\ \;x=wJ_{N}s+GJ_{N}Cs+I_{0} \end{array}$$

where *H* represents the population firing rate function; **s** is the vector representing the average gating variable for each region; *a*, *b*, *d*, and σ are scaling parameters; γ and τ_*s*_ are kinetic parameters; ν is the stochastic input vector; *w* is the local excitatory recurrence; *J*_*N*_ is the synaptic coupling; *G* is the general coupling factor; **C** is the connectivity matrix; *I*_*o*_ is the effective external current; and **x** is the state variable vector for the regions. This model is applied in Deco et al. (2013) to describe a system dominated at the measured time frame by NMDA gating, while AMPA and GABA gating are neglected as “fast” variables. For a complete description and analysis of the model, see Wong and Wang (2006) and Deco et al. (2014).

Here, we apply a mapping from the DMFM to a network of point neurons. In our simulation, each region contains two populations, one excitatory (80% of the total neurons in the region) and one inhibitory (20%). In this case, neurons in NEST do not represent biological neurons but processing units whose mathematical description at population level is equivalent to the elements which comprise the DMFM. The initial parameters used to set up the network are taken from Deco et al. (2013) and detailed in Table 2. For a complete explanation of the model and its motivation, see Deco et al. (2013) and Wong and Wang (2006).

**Table 2:** Network parameters taken from Deco et al. (2013) for each region.

**Parameter**	**Excitatory neurons**	**Inhibitory neurons**
Number of neurons *N*_r_	160	40
Capacitance of the membrane *C*_m_	0.5 nF	0.2 nF
Membrane leak conductance *g*_m_	25 ns	20 ns
Resting potential *V*_L_	−70 mV	−70 mV
Threshold membrane potential *V*_thr_	−50 mV	−50 mV
Reset membrane potential *V*_res_	−55 mV	−55 mV
Refractory time τ_ref_	2 ms	1 ms

Recurrent excitatory connections have a strength of 1.4pA, while recurrent inhibitory connections have a weight of −1.0pA. Each neuron per region initially receives 160 excitatory connections from the local excitatory population. Only inhibitory connections are created during the simulation (blue dashed arrow tagged *J* in the region zoom in of Figure 7A) since we are only interested in substituting the feedback inhibition control algorithm described by Deco et al. (2014). The inter-regional connectivity (black lines between regions in Figure 7A) is specified from structural data obtained by DTI, which results in a connectivity matrix *C*, but further regulated by the general coupling parameter *G*, a multiplicative factor. This enables the linear modification of the strength of the connections without altering the ratio of connectivity among regions. Thus, the total weight of the connections between regions is equal to *G*·*C*pA. Each connection between regions is made between a single representative neuron in each excitatory population. Connections between regions are only excitatory. Additionally, all neurons receive independent background input from a Poisson generator producing spike trains with a rate of 11.9kHz.

We followed the procedure described by Deco et al. (2014) through the generation of synaptic activity, substituting the feedback inhibition control used in that paper with our interactive exploration method. The strength of the background input was tuned to achieve a firing rate of 3spikes/s for the excitatory population and 8spikes/s for the inhibitory population when regions were isolated (without inter-region connections). In Deco et al. (2014), an iterative tuning strategy was used to determine the intra-region inhibition for the DMFMs required to produce an activity profile consistent with experimental observations. The key insight inspiring our approach is that finding the intra-region inhibition can be mapped on to determining the number of inhibitory connections required to produce the same activity pattern in a multi-area spiking neuronal network. Finding the right amount of inhibition per region which satisfies all the dependencies is still a hard multi-objective optimization problem, especially if the space cannot be interactively explored. This is demonstrated in Figure 4, which shows the result of simulating one static parameter setup for the connectivity generation programmatically.

**Figure 8:**
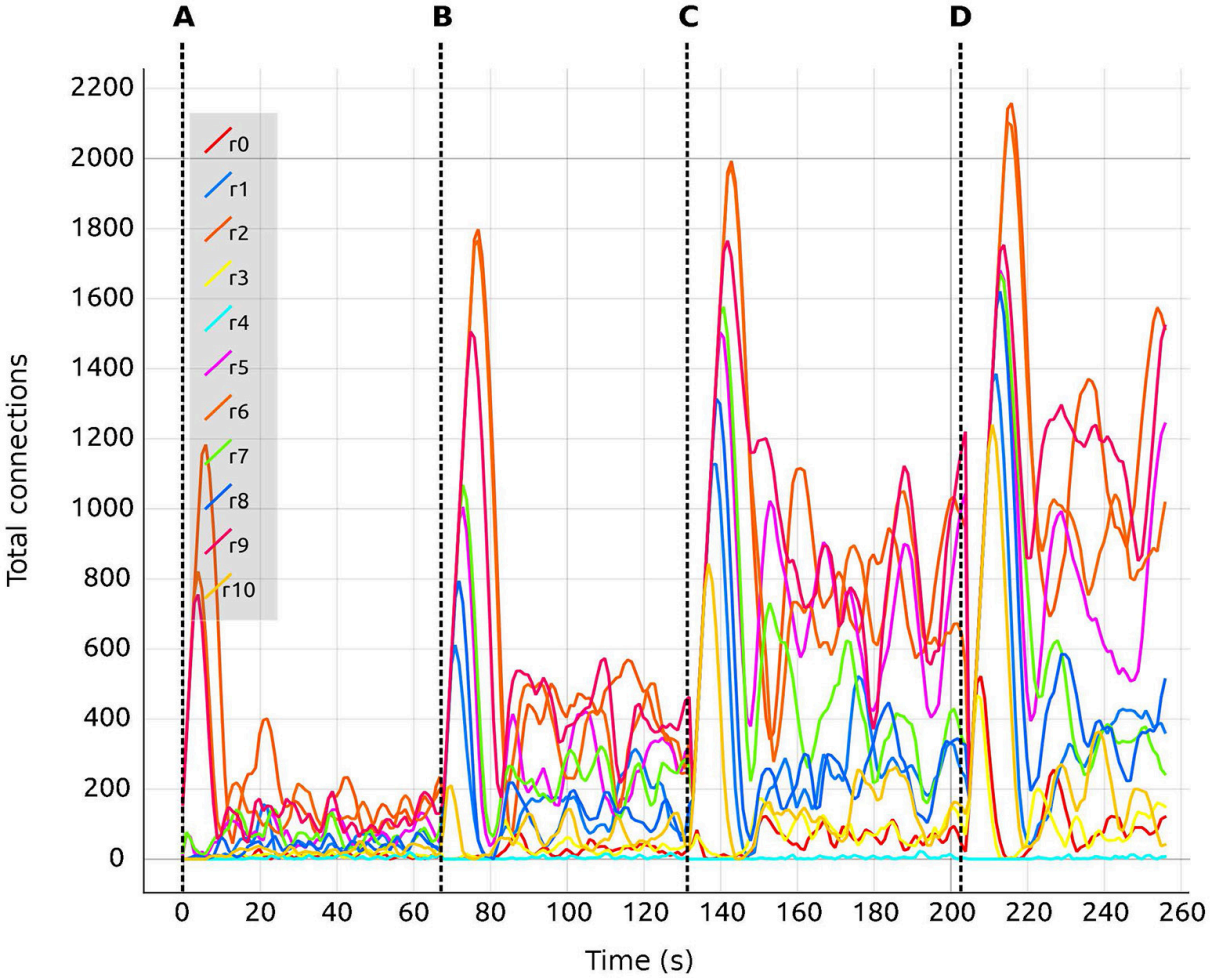
Total number of connections for selected regions (0–10) as a function of biological time. Colors are synchronized between this plot and the *Color Editor*. Vertical dashed lines separate sections of the simulation with differing values of the global connectivity coupling (see section 5), *G* = (A) 0.5; (B) 1.0; (C) 1.5; (D) 2.0. Regions are coupled and numbered from 0 to 67. Tag *rX* identifies the curve for the total number of connections corresponding to the *Xth* region.

In this setup, the tool was used with different values for the inter-region global coupling factor *G*. A complete view of the visualization and steering tool for this use case is shown in Figure 3. By using the tool, we detected that as *G* grows, it becomes more difficult to bring all regions to the desired activity state, and the standard deviation of the average firing rate increases as well. *G* has this impact because any change in one region due to *G* has a strong impact on all other regions dynamically reacting to the change in *G*. This effect is visible in Figures 7, 8, where the time it takes for all regions to stabilize increases as the value of *G* grows.

**Figure 9:**
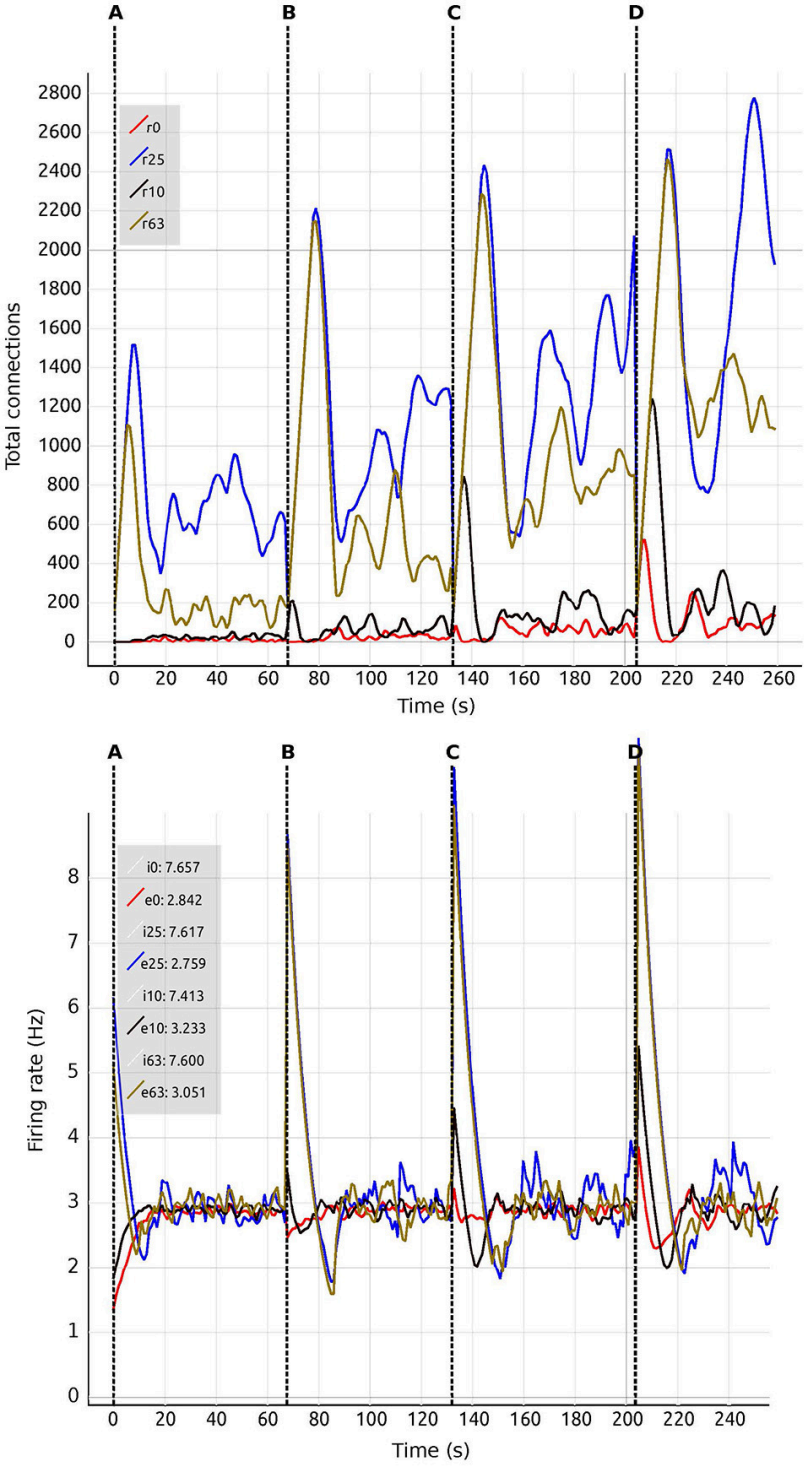
Number of connections **(Top)** and firing rate **(Bottom)** shown in comparison of four regions (0, 10, 25, 63). Vertical dashed lines separate sections of the simulation with differing values of the global connectivity coupling, *G* = (A) 0.5; (B) 1.0; (C) 1.5; (D) 2.0. Regions are numbered from 0 to 67. Tags *eX* and *iX* identify curves for excitatory and inhibitory populations in the *Xth* region. The number at the side of the tags denotes the current value of the average firing rate for each region.

We are also able to detect which regions are more crucial for stability, since they have a higher inter-connectivity to other regions. Figure 9 shows a comparison of the evolution of the firing rate and outgoing connections of four regions. Each peak shows an increment in the global coupling value *G* by 0.5, starting from a base value of 0.5. Regions 25 and 63 show large oscillations due to their high connectivity with multiple other regions. Conversely, regions 0 and 10 rapidly reach a stable state even for high values of *G*. This capacity for detailed inspection allows the researcher to verify that all regions reach the desired average activity while the simulation is running, and thus drastically decreases turn-around times to research this behavior.

**Figure 10:**
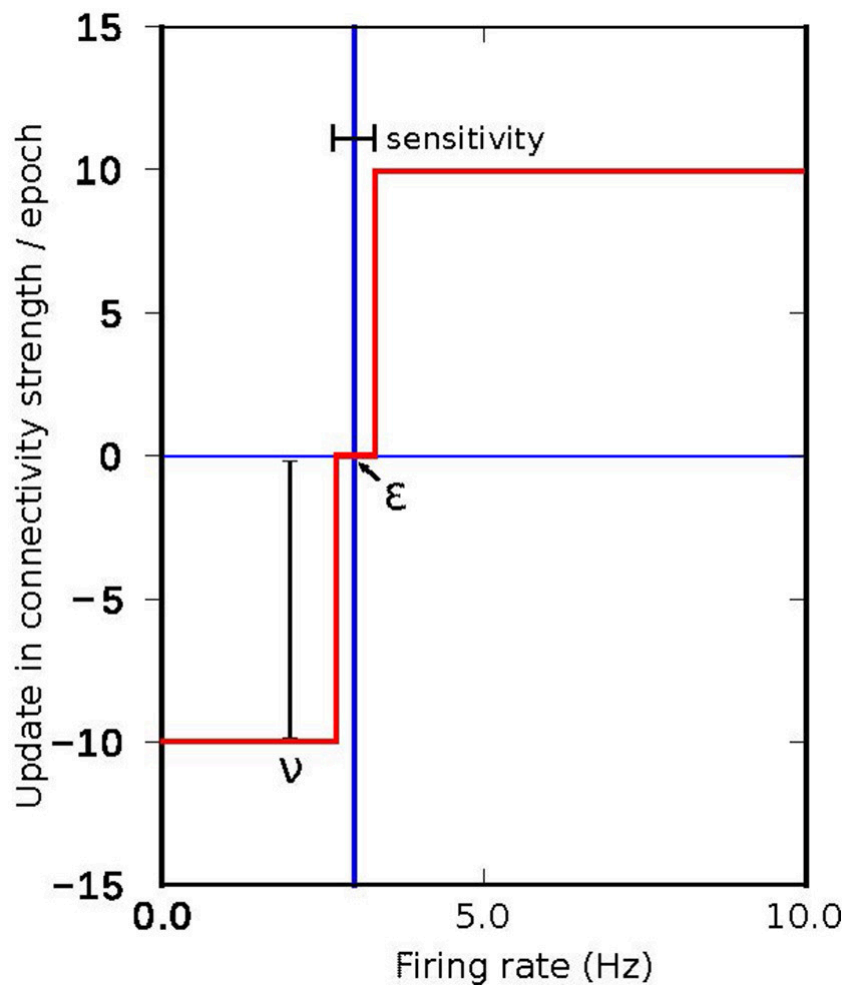
Connectivity update rule employed in the algorithms proposed in Deco et al. (2014) and Schirner et al. (2016), where a fixed value is added or subtracted iteratively to the inner inhibitory connectivity until convergence to the desired firing rate is achieved in each simulated region.

The search algorithms proposed in Deco et al. (2014) and Schirner et al. (2016) are based on an update pattern which (in the same terms as the algorithms used in this work) can be described by a fixed step update around the target activity, as shown in Figure 10. The effectiveness of a fixed search approach in the connectivity parameter space depends mainly on two factors. First, the effectiveness is dependent on the size of the correlation step. If the step is too small, it will take too long to reach the target activity if the initial conditions are not close to the solution. If the step is too large, the system will oscillate because the corrections are too coarse. Second, the effectiveness is dependent on the accuracy. The speed to find a solution is inversely proportional to the desired accuracy. The correction step should also be smaller than the accuracy, otherwise the system may oscillate indefinitely around the final target state without ever reaching a state with the desired accuracy. In summary, the ability of the search algorithm to find a solution depends on the initial conditions, the size of the update step and the desired accuracy. Our proposed approach allows the size of the update step and the speed with which changes take place to be adapted during simulation. This solves the problem of the dependency between step and accuracy and also allows the system to potentially find a solution from a broader range of initial conditions due to the capacity to increase the resolution of the search as the target state is approached.

In addition to the advantages in speed and use of computational resources which our expert-steered approach confers over brute force parameter search (and which may, in fact, be computationally intractable), the process of steering allows the researcher more insight into the system. Whereas in the first use case, the primary finding was that multiple connectivity configurations can result in the same activity profile, in the second use case we are able to identify which regions are most critical for the overall network stability, as illustrated in Figure 9. Thus, interactive visualization can support the researcher in sensitivity analysis, which is essential for understanding the main driving parameters of the model and for making better inferences about the relations between parameters and function. As with the multiple configurations observed in the first use case, it is rare to encounter a network modeling study in computational neuroscience where a sensitivity analysis has been carried out (but see Bos et al., 2016 for a counter example).

### Usage of the tool

In this section, we summarize the main steps required to use the tool to take the system from its initial state to a final connectivity setup where the target mean activity values are achieved. A step-by-step tutorial video of carrying out parameter exploration on a network using our tool is provided in Supplementary Material (Movies 1, 2). In the following, we make reference to the requirements listed in section 4. The first step during the simulation steering is to determine which regions have one or more of the following characteristics (**R2–R5**):

- the electrical activity is far from the target activity, and there is no tendency of the system to correct for this error (or the correction is too slow);
- the electrical activity oscillates around the target activity and the oscillations are of equal or higher amplitude in each cycle;
- or the number of connections does not converge even though electrical activity is around the target activity.

This is achieved using the visualization tool by observing the firing rate and connectivity plots. Figure 7 shows the evolution of the firing rate for the first ten regions of the brain model. Figure 8 shows the changes in connectivity which are guided by the homeostatic growth rules defined for the structural plasticity algorithm. Each curve in the plot is uniquely identified by color and linked to a population or region, thus enabling the assessment of the three above listed characteristics. Reaching the targeted stable state is indicated when all firing rate curves converge to the target activities while the connection curves flatten to horizontal lines. This allows the user to simply and effectively identify which regions deviate from the target state and to correct the structural parameters according to the following criteria:

- If the actual electrical activity is far away from the target activity, the growth rate ν for that region should be increased (**R6**).
- If the actual electrical activity oscillates around the target activity, the growth rate ν for that region should be decreased in small increments and the value of η should be reduced to decrease the rate of change in the number of created and deleted synaptic elements around the target point ε (**R6–R7**).
- If the number of connections does not converge, highly interconnected regions should be identified and the growth rate ν should be modified down in all of them (**R7**). In this case, the update interval can also be modified to a smaller value to have a faster response of the control changes in the connectivity (**R8**). A shorter update interval allows better and smoother control, but impacts the performance of the simulation.

The resulting network state can be saved and used later as a starting point for other parameter combinations, thereby minimizing the need for further computations using similar values of the global coupling term (**R9–R10**).

### Implementing further use cases

Using an event-driven architecture, our framework provides a convenient way for domain scientists to extend the tool to their needs. This tool can be used with any neuron and any synapse model in NEST, except for gap junctions. By using the scripts provided in the Supplementary Material (versions for all use cases discussed in this manuscript) as templates, the user can easily change the neuron and synapse model to explore the impact of these variations. An instrumentation manual which specifies the steps required to integrate the tool with other network models implemented in NEST can be found as part of the Supplementary Material. The instrumentation manual provides instructions based on examples for NEST, but the tool can be adapted to other simulators providing a Python interface by replacing the corresponding functionality. However, if the simulator does not provide an interface to Python, instrumentation will require substantial development effort by the user. Table 3 provides estimates for the complexity of adapting the *nett* messaging library to different use cases. The complete tool and the underlying messaging framework is open source (for further details, see Supplementary Material).

**Table 3:** Estimation on the complexity to adapt the *nett* messaging framework to different steering and visualization use cases.

**Challenge**	**Solution**	**Complexity**
Change network topology	Change number of populations	Simple
Increase the size of the network	Increase the number of neurons in the simulation script	Simple
Retrieve additional parameter	Create new event definition	Medium
Add a new model parameter	Create new event definition	Medium
Connect different simulators with Python interface	Create new event definition for the specific simulation values	Medium-hard
No Python / C++ environment	None	Hard

### Simulating on a supercomputer

To leverage the power of supercomputers to reduce turn-around times for parameter space exploration, the simulation scripts can be adapted to use MPI. In this section, we show an example of adapting the whole brain simulation use case described in section 5.2 to supercomputers. To ensure that each process is in sync with all steering commands, one process (rank 0) serves as master. Only this master process establishes a connection to the visualization front-ends and processes their steering events. Then parameter synchronization is conducted via synchronization barriers with the remaining compute nodes. The master process is responsible for gathering the electrical activities and total connections from all other compute nodes to finally send these to the visualization front-ends.

After all the simulations had been parallelized, we adapted the tool to cope with the supercomputing environment. A challenge of the current usage conditions of most supercomputing environments is their batch-mode operation where users submit jobs which are granted compute time after a possibly long delay; interactive supercomputing is still a work in progress as outlined in Lippert and Orth (2014). Since our tool relies on a network connection to NEST, the IP-address of the compute-node running the simulator is unknown *a priori*. To circumvent this issue, we rely on the supercomputer's global file system: when the simulation is granted compute time, the node's IP-address is obtained and written to disk. Subsequently, all visualization services use this configuration file and connect to the given address. However, one limitation of this approach is the need to start the simulation first.

Since the visualization tools are independent of the network topology and size, the scaling impact of the network's performance can be measured while neglecting the communication overhead. To simulate a larger number of populations with a larger number of neurons, it is crucial to use supercomputers. To this end, we deployed the tool to the JURECA supercomputer at the Jülich Super Computing Centre. JURECA has 260 compute nodes with Intel Xeon E5−2680 v3 Haswell CPUs with 2 × 12 cores per CPU, 128 GB of RAM per node and runs CentOS 7. To assess the speed-up obtained using this machine, we used the third use case's setup and measured the execution times for 50 updates of connectivity in the network, with an update interval of 100 ms. Using a full node on JURECA, we were able to obtain a 2.94-fold speed-up compared to the workstation setup, which uses 8 Intel Core i7−4710MQ CPUs @ 2.50 GHz and 16 GB of RAM running on Ubuntu 16.10.

**Figure 11:**
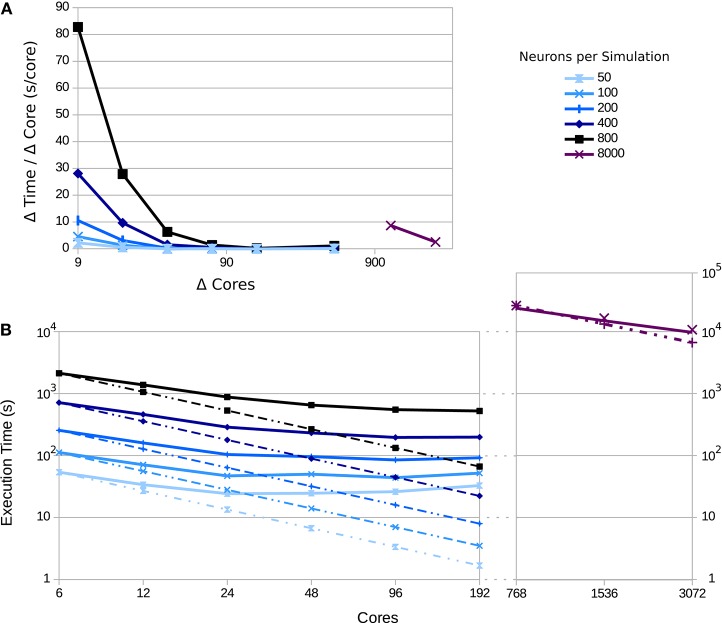
Execution time as a function of the number of compute nodes for varying numbers of neurons per population: 50, 100, 200, 400, 800, and 8, 000; curves shaded from light to dark. Dotted lines indicate ideal scaling, while solid lines represent experimental results. **(A)** The change in time consumed per core added. Each point is the difference in the execution time divided by the difference in cores for consecutive points in **(B)**; points are placed at the midpoint between the source measurements. **(B)** The execution time for each simulation as the number of cores are varied; for the simulation with 8, 000 neurons per populations, measurements were made only for 768–3,072 cores, since fewer cores leads to excessive time demands. The simulated biological time was 5 s using an update interval of 100 ms.

Figure 11 shows a strong scaling test for different numbers of neurons per population. Simulation scalability increases with the number of neurons per population—particularly for 8, 000 neurons per population (a total of 544, 000 neurons in the network). This is due to the network size and spike distribution overhead; larger networks benefit more from the larger number of compute-nodes and overcomes the inter-process communication and intra-process spike distribution overhead up to the point that the global number of spikes dominates performance (for a current discussion, see Jordan et al., 2018). In addition, the number of synapses increases quadratically with the number of neurons per population, which highly impacts the scalability of the simulation.

On the other hand, visualization scalability is dominated by the data gathering step at every update interval. For the case of large networks like the 8, 000 neuron network, the impact of the data gathering step can be reduced by gathering information from only a portion of the network. A well selected statistical sample would provide enough information about the ensemble behavior of the populations while benefiting performance. The current paradigm for the tool funnels data from a large number of compute backends to a single frontend visualizer. In order to scale with increasing numbers of backend nodes for massive supercomputing, a more complex data flow and analysis framework will be needed, such as a multi-node reduction stage to reduce the impedance between the backends and frontend, as well as reducing the load on the fronted. A generalized software framework for such infrastructure to couple visualization with supercomputing at scale is, to our knowledge, currently not available, and is a work in progress.

## Discussion and conclusion

In this paper, we have introduced a visualization and steering tool for the interactive analysis of connectivity generation in NEST. To show its applicability, we have presented two use cases where the tool was used to visualize and steer populations of point spiking neurons to reach a desired target activity level. Our results indicate that by interactively exploring the parameter space and possible trajectories, scientists can gain a better understanding of the system and concentrate on regions of biological interest, as compared to a blind brute force exploration. The improvements over brute force exploration are due to the effects of changes in specific parameters in the network which can be visualized and states outside the admissible regions which can be identified and excluded from further simulation. These improvements lead to a reduction in computational resources and an educated definition of interesting parameters, states and trajectories.

In this work, we have presented results using the interactive steering and visualization tool for two use cases where a desired firing rate was set by the modeler at the beginning of the simulation. We have used firing rate calculations produced internally in NEST to guide the generation of connectivity. This method for computing the mean firing rate can impact the performance of the control system since controllability depends on the delay between measuring an observable and producing a response. However, the tool is independent of this calculation and other techniques, such as spike train binning, can be used instead to increase the controllability. Calculation of firing rates on streams of spike trains might become computationally intensive with increasing network size. Future implementation of other techniques to increase the data gathering speed will lower delays and allow other spike processing techniques to be efficiently implemented as alternatives to the convolution approach.

The use cases presented were selected for their differing degrees of complexity in terms of connectivity and network definition. In the simple use case, different connectivity configurations lead to the same activity profiles even when some of the trajectories are biologically inadmissible. With our approach, the user can concentrate on exploring only those configurations which are of interest in answering the scientific question posed. In Figure 6, we show different trajectories produced using structural plasticity following homeostatic rules to fit the system to a firing rate profile. Even the non-biological parameters of the optimizing algorithm itself have an impact on the final configuration of the network. For example, if one performs a gradient descent to optimize the activity profile of a network, the results will be sensitive to any arbitrary choice of initial states of the populations and connectivity. With our tool we can characterize the distribution of representative models and results.

In the second use case, our tool also enables a sensitivity analysis of the system by visualizing the effect that changes in the connectivity have on the dynamics of the full system. Thus, the user can draw better conclusions about the relationships between the controllable parameters, in this case connectivity, and the observables of the system, in this case the firing rate of each population. We can see the relative sensitivity of the system to the biologically relevant parameters (connectivity) and the non biological parameters of the optimization algorithm. Our tool can provide more insight into how different types of synapses are created or modified in the neural circuit to give rise to different features in the dynamics of the system.

As we have discussed in this paper, a brute force exploration of the parameters of a network can easily become a computationally intractable problem. Choosing a single random configuration or even only a small sample of configurations from the whole space without a proper characterization of their distribution is unlikely to lead to a statistically valid distribution of the results. Interactive visualization is a way to move toward statistically validated conclusions as it allows an assessment of the essential features of the system, ultimately leading to automated sampling.

While the resulting connectivity patterns are not necessarily unique, our approach enables exploration and assessment of these solutions and their paths. The main contribution of this approach is the use of interactive visualization and parameter control techniques. These techniques allow the system to be controlled and stabilized within a physiological configuration space by an expert. When increasing the number of neurons and populations, the number of parameters to tune increases, resulting in an ever harder-to-reach stable state. Thus, interactive visualization becomes even more important. The knowledge gained through interactive exploration can lead to the development of automated tools assisting in the parameter space exploration.

Using this approach, we can reduce the turn-around times of exploring different connectivity configurations in comparison to simulating all possible parameter configurations and assess reasonable configurations in a later phase. The speed-up achieved by this exploration is mainly due to four factors. First, it is not necessary to simulate the system for long times iteratively; instead, the modifications are performed on demand. Second, partial solutions can be reused for different global parameter combinations, resulting in the reduction of total computational costs. Third, the user can visualize the behavior of the system's observables with respect to individual parameters, allowing the user to isolate regions of interest and form a better understanding. Finally, we can study the transition points in the activity of the networks, which are produced by the underlying connectivity variations, and interact with the tuning algorithms by visualizing their impact.

As stated before, the connectivity solutions and paths to solutions for the presented use cases are not unique, rendering a knowledgeable exploration process crucial. Thus, the interactive analysis process can help the user accomplish the following:

1. Form an understanding of the implication of different parameter setups for each network model.
2. Validate the models.
3. Define biologically meaningful populations of interest for the simulation.
4. Derive measures for the automatic or semi-automatic assessment of the models' behavior leading to automated tools guiding the exploration process.

While the generation of connectivity based on empirical constraints for the dynamic system or experimental data inherently leads to non-unique solutions and especially solutions which are physiologically implausible, the ability to identify and explore subsets of the solution space is valuable to form an understanding of the dynamic nature of these systems.

In this work, we have formalized the effects of dynamic connectivity of a network in terms of control theory. We take into account that the network starts at an initial state and is taken to a final state through the introduction of control signals which alter the connectivity of the network. In this case, control of the synapse creation and deletion is induced by the structural plasticity algorithm. The eigenvalues of the Liouvillian of the network are thus modified with these signals through the evolution of the simulation and the state of the neurons in the network is changed. Visualization shows the immediate effects of the control signals in the system. The results shown in section 5.1 exemplify how even a simple network can traverse different admissible trajectories (Figures 6A–E) using different elements from the set of all possible controls. We show how the unconstrained system can traverse an inadmissible trajectory (Figure 6F) or end in states outside of the admissible set (Figure 6D). We have also seen how inadmissible control signals are still able to give rise to admissible trajectories and final states (Figure 6E).

We adapted the tool to scale with supercomputers allowing larger networks to be simulated and finer simulation stepping to be used, thus achieving more accurate results. This way, researchers can explore the manifold solutions and paths of connectivity satisfying average activity targets in a variety of neural network models. Our tool gathers data from the simulation at specific intervals, which impacts the performance as the networks become larger. Continuously streaming data from of the simulation by using, for example, MUSIC (Djurfeldt et al., 2010) or the NEST I/O backends can reduce this bottleneck and allow greater flexibility in the network size.

In summary, our interactive tool provides the means to visualize and steer connectivity generation of a running NEST simulation to stabilize complex non-linear systems. The applied concepts of the tool are generalizable and extensible to other types of systems with similarly large degrees of freedom. Adapting and exploring further model parameters, e.g., synaptic weights and delays, background input frequency, and variation in weights of spike-timing-dependent plasticity synapses is possible. Our implementation is open to the public (see Supplementary Material).

In the future, we would like to explore further techniques to track already explored parameter spaces, to develop semi-automatic systems to guide researchers in tracking manifold solution spaces and to extend the tool to support further use cases. Currently, the saved state refers only to the connectivity and last values of all variables at the time of saving. We are working to provide a visualization that shows parameter changes for reproducing all trajectories. For the moment, the loading features are limited and the subject of future work. In addition, we are adding support for machine learning algorithms coupled with the interactive exploration for various network variables beyond connectivity. Our goal is to to detect oscillations and other troubling behavior in the network using machine learning and then to correct this behavior by use of the controllers. Other target control measures such as power-spectrum shape and inter-population correlations may be interesting as complex control variables in the context of machine learning. The modularity of the software, primarily derived from applying an event driven design, allows for such additions in a non-intrusive manner.

Linking the time axes of the activity and connection plots to allow for coordinated zooming is currently not supported but would be a useful extension to the analysis workflow. A visualization of changes in the network's eigenvalues as connectivity evolves is also subject to future work. The creation of additional plots for further variables is simple and can be achieved by adapting the scripts used in the presented use cases (see Supplementary Material). Connecting another visualization application to the NEST simulator is in principle feasible but requires adapting the visualizer to our communication protocol.

We argue that it is crucial to explore the distribution of paths to solutions instead of focusing on just *a* possible solution satisfying a set of constraints. To develop this understanding, interactive exploration of dynamic systems is a key tool for developing mathematical intuition, and thus for deriving mathematically robust descriptions. These descriptions are then amenable to further automated investigation of characteristic solution ensembles.

## Author contributions

CN and SD-P have contributed equally to this paper. CN developed the interactive steering tool and the framework. In addition, he designed the data flow for steering the main structural plasticity parameters. SD-P, AP, and AM defined the use cases. SD-P evaluated the results and compared the process of parameter navigation with and without the steering tool. AP provided the high performance computing knowledge to port and optimize the code for supercomputing usage. BW, BH, and TK provided scientific guidance on the visualization tool. AM provided neuroscientific guidance and assessed the usability of the tool for generalized use cases. CN, SD-P, AM, and AP wrote the paper.

### Conflict of interest statement

The authors declare that the research was conducted in the absence of any commercial or financial relationships that could be construed as a potential conflict of interest.

## Supplementary material

Movie 1 Introduction to the interactive steering and visualization tool.

Movie 2 Using the interactive steering and visualization tool in a specific use case for structural plasticity in NEST.

Presentation 1 Instrumentation manual.

We have included two videos as Supplemental Material that demonstrate our visualization tool. The first video presents its basic structuring and functionality. The second one discusses one use case for structural plasticity and explains how the tool can help in the assessment of the simulation. Moreover, we have included an instrumentation manual describing the general process for modifying simulation scripts in order to use the tool.

The *nett* messaging framework can be downloaded at https://devhub.vr.rwth-aachen.de/VR-Group/nett.git, and https://devhub.vr.rwth-aachen.de/VR-Group/nett-python.git. The NEST structural plasticity framework can be found at https://github.com/sdiazpier/nest-simulator.git, branch sp_rate and tag sp_viz_rate.

The visualization tool, simulation scripts for both use cases, and a user manual to build the code can be found at https://github.com/sdiazpier/isv_neuroscience. In addition to the use cases discussed in this work, a script showing the instrumentation of the cortical microcircuit model (Potjans and Diesmann, 2014) is also provided in the aforementioned git repository.
