# Supplementary material for: Toward Rigorous Parameterization of Underconstrained Neural Network Models Through Interactive Visualization and Steering of Connectivity Generation
Source: Front Neuroinform. 2018 Jun 1;12:32. doi: 10.3389/fninf.2018.00032 (PMC5992991; doi:10.3389/fninf.2018.00032)
Supplement: Presentation 1 — Instrumentation manual. [file Presentation_1.PDF]

Instrumentation manual for the interactive steering and visualization tool

March 26, 2018

1 Introduction

This document will explain a general methodology to integrate neuroscience simulation scripts written in Python with our Interactive Steering and Visualization tool. This tool is independent of the simulation platform, so all instructions will be kept generic. We will not focus on the installation of the tool in this document. For details on this please refer to the installation manual.

2 Preparation

1. Identify the variables to be observed or monitored from your simulation and how you collect this data in your Python script.
2. Identify the variables you want to control or modify in your simulation and how you can inform these changes to your simulator.
3. Identify the range of values that the variables can have.
4. Define the structure of your network in such a way that you can clearly identify populations. Observable and controllable variables should be linked to populations. In this manual we will specifically focus our attention to steering and visualization of collective variables, mostly average values inside each population. However, the tool is able of monitoring more finely grained variables too.

As explained in the manuscript this interactive steering and visualization framework consists of several modules. For the use cases depicted in the manuscript, these modules are implemented in Python files as follows:

1. Control panel: simulator_controller.py
2. Manipulation of Structural Plasticity Parameters: eta_manipulator.py and growth_rate_manipulator.py
3. Activity plot: fr_plotter.py
4. Connectivity plot: connection_plotter.py
5. Color Editor: region_color_selector.py
6. Region selector: region_selector.py
7. Helper files: helper.py

3 Importing the necessary modules

In the main script, it is important to import the helper file as it gives access to all the communication functions to collect and send information between the simulation script and the tool.

```
from helper_simple import *
```

4 Redefining the simulation process

In this framework, we consider interactivity with an on going simulation. This means that we need to redefine the way the simulation takes place. The easiest way to do this is to fragment the simulation in small units of time and perform the simulations in an infinite cycle. After each of the simulation steps is finished, information about the results of the

simulation can be collected and visualized. This also makes space to collect user changes in the controllable variables and send these changes to the simulator.

Below is an example of how a linear simulation could look like. Here, a 'run' function has been defined to encapsulate the simulation steps.

```
def run():
    s = Simulation()
    s.prepare_simulation()
    s.create_nodes()
    s.connect_nodes()
    s.simulate(simulation_time)
```

Below is an example of how the linear simulation script has been included in an infinite cycle, where short simulations of length 'record_interval' are performed iterably while a pause or quit signal is not received from the user.

```
def run():
    s = Simulation()
    s.prepare_simulation()
    s.create_nodes()
    s.connect_nodes()
    while s.get_quit_state() == False:
        while s.get_pause_state() == False:
            self.update_controllables()
            nest.Simulate(self.record_interval)
            self.record_observables()
```

5 Setting up the communication ports

Our scripts include a function called setup_nett. This function takes care of starting the communication framework called nett and establish the input and output channels for the tool. It can be used as a base when starting the instrumentation process.

```
def setup_nett(self):
  #Only needs to happen on rank 0 in MPI setup
  if nest.Rank() == 0:
      #nett already initialized?
      try:
          current_ip = socket.gethostbyname(socket.gethostname())
          current_ip = '127.0.0.1'
          f = open('ip_address_compute'+'.bin', "wb")
          f.write(str(current_ip))
          f.close()
          nett.initialize('tcp://'+str(current_ip)+':8000')
      except RuntimeError:
          pass
```

Please note that at this point we save the ip of the machine where the simulation process is running. We also define a port, in this case 8000, which should be also used by the visualization modules to connect to the simulation. Please refer to section 6.2.

Now that the communication framework is initialized, we can start creating input and output ports to send and receive information between the simulation, the visualization modules and the steering modules. Below is an example taken from the first use case discussed in the manuscript. It includes setup for output ports for the firing rate, total connections and number of regions. It also includes input ports for a quit command, a pause command, a save command, the changes in the update interval, in the growth rate and in the value of eta.

```
      self.fr_e_slot_out = nett.slot_out_float_vector_message('fr_e')
      self.fr_i_slot_out = nett.slot_out_float_vector_message('fr_i')
      self.total_connections_slot_out =
            nett.slot_out_float_vector_message('total_connections_i')
      self.total_connections_e_slot_out =
            nett.slot_out_float_vector_message('total_connections_e')
      self.num_regions_slot_out = nett.slot_out_float_message('num_regions')
```

```python
        self.run_slot_in = nett.slot_in_float_message()
        self.quit_slot_in = nett.slot_in_float_message()
        self.pause_slot_in = nett.slot_in_float_message()
        self.update_interval_slot_in = nett.slot_in_float_message()
        self.save_slot_in = nett.slot_in_float_message()

        self.quit_slot_in.connect('tcp://127.0.0.1:2003', 'quit')
        self.pause_slot_in.connect('tcp://127.0.0.1:2003', 'pause')
        self.update_interval_slot_in.connect(
                                    'tcp://127.0.0.1:2003',
                                    'update_interval')
        self.save_slot_in.connect('tcp://127.0.0.1:2003', 'save')

        self.observe_quit_slot = observe_slot(self.quit_slot_in, float_message())
        self.observe_quit_slot.start()
        self.observe_pause_slot = observe_slot(self.pause_slot_in, float_message())
        self.observe_pause_slot.start()
        self.observe_update_interval_slot = observe_slot(
                                              self.update_interval_slot_in,
                                              float_message())
        self.observe_update_interval_slot.start()
        self.observe_save_slot = observe_slot(self.save_slot_in, float_message())
        self.observe_save_slot.start()

        self.observe_growth_rate_slot = observe_growth_rate_slot(self.regions)
        self.observe_growth_rate_slot.start()

        self.observe_eta_slot = observe_eta_slot(self.regions)
        self.observe_eta_slot.start()
```

For example, we can see that nett.slot_out_float_vector_message(´fr_e´) is used to create an output slot with the label ´fr_e
On the other hand, nett.slot_in_float_message() is used to create an input slot.

6 Gathering observable data and visualizing it

Gathering information about the observables and sending it to the visualization modules involves setup in the main script and setup in the visualization module.

6.1 In the main script file

We can now write a function to collect the values at each point of time in the simulation for each of our observable variables. In this framework, information is transfered among modules via messages. The concept here will be to create a message for each of your observable data vectors. Nett offers different types of vector messages. For example, in the use cases shown in the manuscript, float values are transfered and visualized.

Following is an example of how to create a float vector message for two observable variables.

```python
def record_variables(self):
    if nest.Rank() == 0:
        msg_variable1 = float_vector_message()
```

The next step is to gather the data and add it to the message. This step depends on the Python interface between the script and the simulator platform you are using.

An example of how we do this in our use cases is seen below for NEST, where the firing rate of all the populations is collected and the average is calculated.

```python
    for x in range(0,self.populations) :
        fr_e = nest.GetStatus(self.loc_e[x], 'fr'),  # Firing rate
        fr_e = self.comm.gather(fr_e, root=0) # Multiple MPI processes
        if nest.Rank() == 0:
            mean = numpy.mean(list(fr_e))
```

Once the new values are collected from the simulator, they can be appended to the corresponding message data structure.

```
            msg_variable1.value.append(value)
```

Finally we ship the data in the message structure using the nett framework through the corresponging output slot defined in Sec 5.

```
            self.variable1_slot_out.send(msg_variable1.SerializeToString())
```

6.2 In the visualization module files

In the example scripts we have included two visualization modules, one for plotting the firing rate and one for plotting the connections. The relevant section in this scripts related to the collection of information from the simulation is encapsulated in a class called monitor_feed. This class initializes the ports to receive data from the simulation.

```
class monitor_feed(QtCore.QThread):
  def __init__(self):
    QtCore.QThread.__init__(self)
    self.signal_fr_e = QtCore.SIGNAL("signal_e")
    self.fr_e_slot_in = nett.slot_in_float_vector_message()
    ip = helper.obtain_ip_address_compute()
    self.fr_e_slot_in.connect('tcp://'+ip+':8000', 'fr_e')
```

It is important that the port used in the connect function matches the port provided on the main script for the out slot. In this example, the port is 8000. We store the ip of the simulation process in a file and retrieve this value using the óbtain_ip_address_computeḿfunction from the helper. This is useful when the simulation is running on a different machine as the visualization module, as it happens when running the simulation on a supercomputer.

When an instance is started, it collects data continuously from the port and refreshes the view in the plot. In order to refresh the view, it emits a signal with a particular label linked to the data received.

```
  def run(self):
    msg = float_vector_message()
    while True:
        msg.ParseFromString(self.fr_e_slot_in.receive())
        self.emit(self.signal_fr_e, msg )
```

We will not discuss here how the module interprets the signal and updates the plot, as this is part of its implementation.

7 Gathering changes in variables and propagating them to the simulator

7.1 In the main script file

Here we will do the reverse process, we will gather the changes in the values of our controllable variables and send these changes to the simulator.

First we collect the message containing the changes incoming from the manipulator modules.

```
  controllable1_dict = self.observe_controllable1_slot.controllable1_dict
```

Afterwards we can send this information to the simulator. This, again, depends on the Python interface between the script and the simulation platform. An example of how we do this in our use cases is seen below, where the changes in the growth rate of synaptic elements is provided to the nest simulator.

```
  for x in range(0, self.populations) :
      synaptic_elements_i = { 'growth_rate': -growth_rate_dict[x], }
      nest.SetStatus(self.nodes_e[x], 'synaptic_elements_param',
                                      {'Den_in'+str(x): synaptic_elements_i})
```

7.2 In the steering module files

Here we will take as an example the growth rate manipulator file. First important element here is the initalization of nett. This takes place in the main function as

```
nett.initialize('tcp://' + str(ip) + ':2006')
```

Please note that the port defined here as 2006 must correspond to the port defined as input port in the helper file (see section 7.3).

The class GrowthRateManipulator take care of collecting and sending the changes made by the user to the growth rate variable. During initialization it defines an output port to send the data.

```
self.growth_rate_slot_out = nett.slot_out_float_vector_message('growth_rate')
```

The GrowthRateManipulator class has a function which takes care of getting the new values for the variables and sending them as messages to the correspoding port defined by the simulation script.

```
def send_rates(self):
    msg = float_vector_message()
    for key in self.growth_rate_dict:
      msg.value.append(self.growth_rate_dict[key])
    self.growth_rate_slot_out.send(msg.SerializeToString())
```

7.3 The helper file

The helper file is an essential element for configuration of the tool. The most relevant elements to consider are: The class óbserve_growth_rate_slotánd the class óbserve_eta_slot? These two classes define the points of contact between the steering modules and the simulation. For example, the óbserve_growth_rate_slotćlass initializes the input slot to receive changes in the values of the growth rate as:

```
self.slot.connect('tcp://'+ip+':2006', 'growth_rate')
```

Please note that here the example port 2006 is the same as defined in the corresponding steering module (see section 7.2. The class also has a function which runs on a thread and indefinitely waits for new values to arrive from the steering module.

```
def run(self):
    msg = float_vector_message()
    while True:
      msg.ParseFromString( self.slot.receive() )
      if msg.value != None:
        self.last_message = msg.value
      for x in range(0, len(msg.value)):
        self.growth_rate_dict[x] = msg.value[x]
      self.last_message = msg
```

For more information about nett and the implementation please refer to the wiki page of the nett software: `https://devhub.vr.rwth-aachen.de/VR-Group/nett/wikis/home`.
